# Carbon nanotube based transparent conductive films: progress, challenges, and perspectives

**DOI:** 10.1080/14686996.2016.1214526

**Published:** 2016-09-02

**Authors:** Ying Zhou, Reiko Azumi

**Affiliations:** ^a^ Photonics and Electronics Research Institute, National Institute of Advanced Industrial Science and Technology, Tsukuba, Japan

**Keywords:** Carbon nanotube, transparent conductive film, doping, organic photovoltaics, sensing devices, 40 Optical, magnetic and electronic device materials, 104 Carbon and related materials

## Abstract

Developments in the manufacturing technology of low-cost, high-quality carbon nanotubes (CNTs) are leading to increased industrial applications for this remarkable material. One of the most promising applications, CNT based transparent conductive films (TCFs), are an alternative technology in future electronics to replace traditional TCFs, which use indium tin oxide. Despite significant price competition among various TCFs, CNT-based TCFs have good potential for use in emerging flexible, stretchable and wearable optoelectronics. In this review, we summarize the recent progress in the fabrication, properties, stability and applications of CNT-based TCFs. The challenges of current CNT-based TCFs for industrial use, in comparison with other TCFs, are considered. We also discuss the potential of CNT-based TCFs, and give some possible strategies to reduce the production cost and improve their conductivity and transparency.

## Graphical abstract

**Figure:**
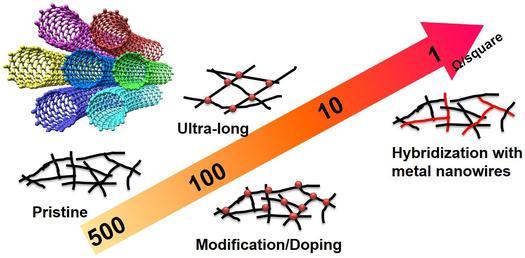

## 1. Introduction

Transparent conductive films (TCFs) are films of optically transparent and electrically conductive materials. Currently, indium tin oxide (ITO) is the dominant material used for industrial-scale TCF application. ITO not only has excellent properties with a sheet resistance of 10 Ω/square at around 90% optical transmittance, but also exhibits outstanding stability and compatibility with both wet and dry device processes. However, future optoelectronics require TCF materials which are mechanically flexible, lightweight and low fabrication cost.[1–5] The growing demand for ITO due to the development of solar cells may lead to an increase in substantial cost because of the relatively rare element of indium. In addition, ITO suffers from poor mechanical flexibility, which suppresses its application for emerging flexible, stretchable and wearable electronic applications. These significant limitations of ITO have been motivating the search for alternative TCF materials for industrial use including novel metal oxides, conducting polymers, metal nanowires, metal grids and carbon nanomaterials. The alternative metal oxides can use abundant materials such as SnO_2_:F, CuO_2_:Al and ZnO:Al.[6–8] Similar to ITO, TCF fabrication of these oxides requires a vacuum and/or high temperature process to achieve high transmittance and low sheet resistance. Apparently the price of ITO TCFs is dominated by the cost of mass production rather than the cost of indium. Solution processed oxide TCFs have been attracting interest as a low-cost technology.[9]

TCFs can be fabricated with poly(3,4-ethylenedioxythiophene):poly(styrenesulfonate) (PEDOT:PSS), a conducting polymer which has also been widely investigated as a hole transport layer for organic photovoltaic (OPV) and organic light-emitting diode (OLED) devices.[10,11] Solution-processed PEDOT:PSS films have been demonstrated to show a sheet resistance of less than 200 Ω/square at above 80% optical transmittance, while they also exhibit poor thermal stability and noticeable blue color.[12–15]

Metal nanowires are becoming a strong alternative to ITO.[16–20] Due to the high conductivity, the nanoscale network of silver nanowires (Ag NWs) can obtain a TCF with sheet resistance of 20 Ω/square at transmittance of 95%, which is even better than ITO. Although silver is more expensive than indium, Ag NWs allow a solution-based roll-to-roll technology, which significantly reduces the overall cost for mass production of TCFs. It has been reported that Ag NW TCFs are used as touch sensor for Lenovo computers (China).[20] However, the thermal stability and chemical stability of metal nanowires need to be further investigated. Copper nanowires (Cu NWs) are becoming a more promising TCF technology, because copper is 100 times cheaper than silver. The best TCFs using Cu NWs show a sheet resistance of 100 Ω/square at transmittance of 95%. The stability of Cu NWs against oxidization is a challenge.[21,22]

Graphene exhibits very good electrical and optical properties in theory, and thus it can be an ideal material for TCF application. However, the electrical properties strongly depend on the quality of graphene.[23–30] The best TCFs using four or five layers of mechanically exfoliated graphene show a low sheet resistance of 8.8 Ω/square at transmittance of 84%.[31] Solution processed graphene TCFs generally show a sheet resistance of above 1000 Ω/square at transmittance of 85%.[32–35]

Carbon nanotube (CNT) thin films with thickness in the range of 1–100 nm can exhibit high electrical conductivity and high optical transparency. Compared with other potential TCF materials to replace ITO, CNT films not only enable an easier fabrication process, but also provide a more stretchable and flexible platform with stronger mechanical strength. The continual progress in the massive production, purification, dispersion and film deposition processes of CNT are leading to a very competitive performance of CNT-based TCFs.[36–50] However, there still remain challenges to further improve the electrical conductivity and stability of CNT-based TCFs. Efforts are also being made to find new markets for CNTs and discover new and promising applications by taking advantage of their stretching and folding properties, etc. In this paper, we focus on the recent progress in fabrication and modification of CNT-based TCFs, and discuss the stability of CNT-based TCFs in detail. We also highlight some examples of CNT-based TCFs in sensing devices, OPV and OLED devices. Finally, we give a summary and analysis on the prospects of CNT-based TCFs.

## 2. Fabrication of CNT-based TCFs

In 2004, the first CNT-based TCFs were fabricated by Wu et al. [51] and Saran et al. [52] by using filtration-transfer and dip-coating methods, respectively. Thousands of papers have since been published on this topic. CNT-based TCFs have been considered as the most promising candidate to replace ITO. CNT-based TCFs can be fabricated by dry or wet processes, where the major difference is whether or not a dispersant is used.

### 2.1. Dry processes

**Figure 1.**
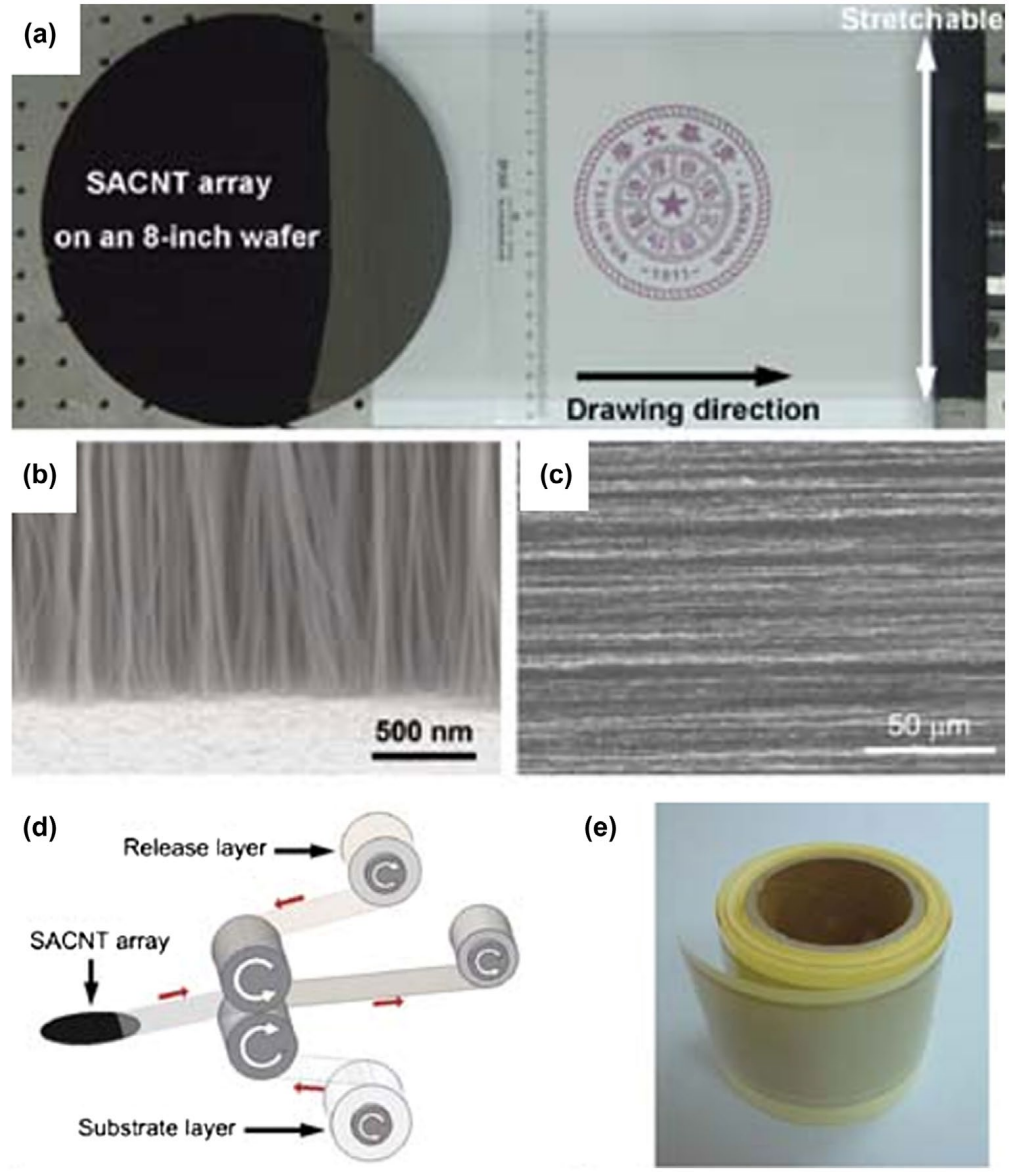
(a) Freestanding CNT film drawn out from a 230-μm high superaligned CNT (SACNT) array on an 200 mm silicon wafer. (b) Scanning electron microscopy (SEM) image of the CNT array on the silicon wafer in side view. (c) SEM image of the CNT film in top view. (d) Illustration of the roll-to-roll setup for producing composite TCFs. (e) A reel of CNT/PE composite TCF produced by the roll-to-roll setup. (Reprinted with permission from [44], copyright 2011 Wiley-VCH.)

CNTs can be produced by chemical vapor deposition (CVD), laser ablation and arc-discharge methods. Generally, for dry processes, CVD is modified to directly grow the CNT film, or transfer the CNT aerosol to film.[53–60] As a result, dry-processed CNT films exhibit higher quality with better separated individual CNTs, fewer defects and better CNT–CNT contact, compared with solution processes. It has been reported that dry-processed CNT-TCF showed a sheet resistance of 84 Ω/square at 90% transmittance.[61] Moreover, a developed CVD technology can fabricate a carbon nanobud (CNB: combining CNT and fullerene) film, which exhibited 150 Ω/square at 97% transmittance.[62] In 2010, Feng et al. [44] demonstrated a straightforward roll-to-roll process to make flexible and stretchable multi-walled CNT TCFs, as shown in Figure 1. They developed a drawing process, which converted the vertical alignment of CNTs in 200 mm silicon wafer into horizontal alignment, directly forming a freestanding, ultrathin, lightweight, transparent, and conductive CNT film. A roll-to-roll process was utilized to fabricate CNT/polymer-sheet composite film. Further doping with vacuum-evaporated metal films greatly decreased the resistance. In this work, CNT-based TCFs exhibited sheet resistances of 208 and 24 Ω/square at 90% and 83.4% transmittance, respectively. The use of special super-aligned CNT arrays limits its wide application, while this technique has been proved to be a promising route to CNT-based TCFs easily, effectively and cheaply.

### 2.2. Wet processes

**Figure 2.**
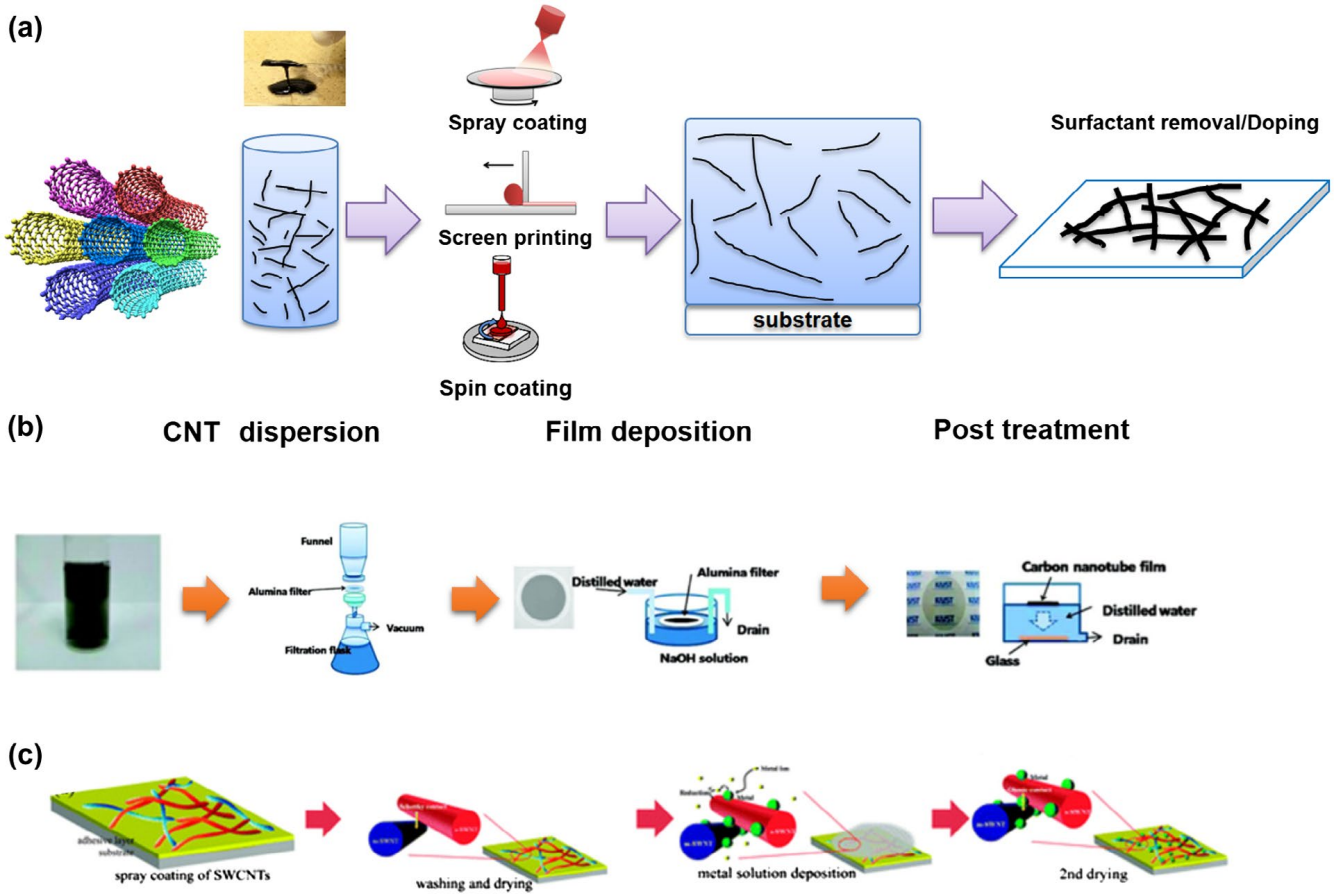
(a) Schematic of typical wet process for fabricating CNT-based TCFs, which includes CNT dispersion, film deposition and post treatment (surfactant removal and/or doping). Two examples for fabricating CNT-based TCFs: (b) vacuum filtration and alumina membrane transfer to make CNT films (reprinted with permission from [80] Copyright 2010 American Chemical Society); and (c) spray coating and post treatments of CNT films (reprinted with permission from [101], copyright 2010 Royal Society of Chemistry.)

Wet processed CNT-based TCFs are also competitive for industrial use. This technology enables a low temperature process, where a vacuum is not necessary. Thus, the production costs can be greatly reduced. Moreover, it broadens the substrate selection. Plastic or other non-traditional substrates can be used. Figure 2 gives the typical procedures for wet processes: (1) preparation of CNT dispersion; (2) film deposition; and (3) post treatments including removal of surfactant and/or doping. The properties of CNT-based TCFs depend on the quality of CNT materials, quality of CNT dispersion, morphology of CNT film, dispersant, and doping treatment. Raw CNTs are in the form of a black powder. Due to their large aspect ratio, large surface area and strong van der Waals interaction, CNTs strongly stick together to form large bundles. There have been many research reports and reviews on the preparation of CNT dispersion.[63–80] However, it remains a challenge to disperse ultra-long CNTs (above 10 μm) without damage or shortening. Three major methods can be summarized to disperse CNTs in liquid solvents: (1) direct dispersion of pristine CNTs in organic solvent; (2) dispersion of covalently functionalized CNTs; (3) dispersion of pristine CNTs with aid of dispersants such as surfactants or polymers. Although use of dispersants increases the processing steps, and residual dispersants increase the contact resistance between CNTs, dispersant-aid CNT dispersion is the most widely used method due to the significant advantages for industrial use. We mainly review dispersant-aid CNT ink for TCF applications. Among a number of studied surfactants, those most commonly used for TCF applications are ionic sodium cholate (SC), sodium dodecyl sulfate (SDS), sodium dodecyl benzene sulfonate (SDBS) and non-ionic Triton X-100. These surfactants enable high CNT concentrations of up to 20 mg ml^–1^ in aqueous solvent. Low concentrations of the surfactants will not affect the electrical conductivity, while the viscosity of CNT ink is difficult to control. Polymers can be used to fabricate dispersion by wrapping CNTs. By choosing suitable polymers, such as cellulose derivatives, surface tension as well as viscosity of the dispersion can be widely modified for various deposition processes from spin coating to screen printing. However, polymer-assisted dispersion is not widely reported for TCF applications because it is difficult to remove the insulating polymer after film deposition. High-temperature annealing or long-term solution process are usually needed to remove the polymer. Therefore, it may not be applicable for plastic substrate or for mass production.

**Figure 3.**
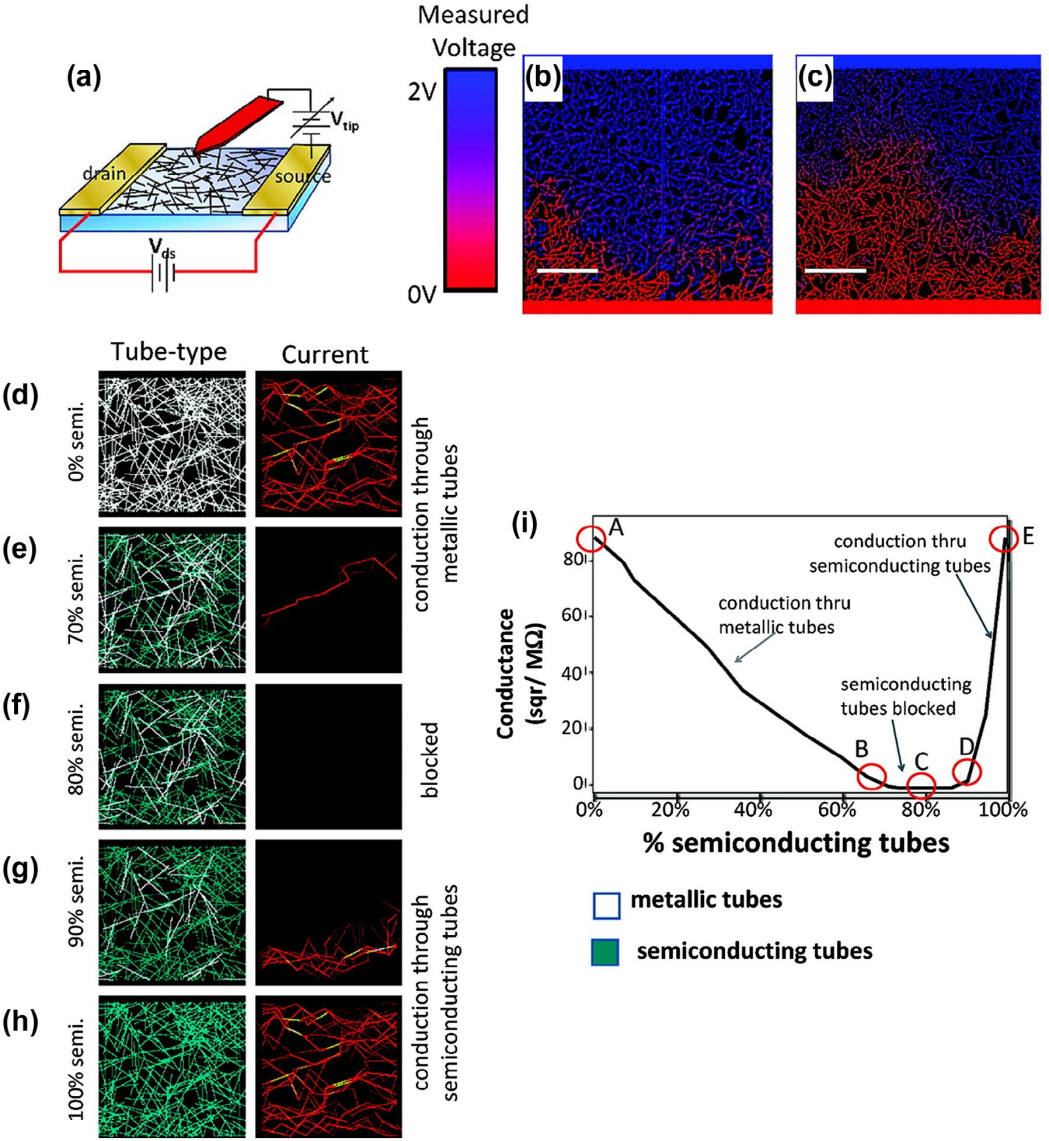
(a) Schematic showing the measurement technique used to image voltage drops in CNT films. (b, c) Experimental images on nominally identical devices. (d–h) Current flows as the ratio of metallic (white) and semiconducting (green) tubes is varied. (i) Expected conductance as a function of proportion of semiconducting tubes (assuming for simplicity that an unblocked semiconducting tube conducts as well as a metallic tube. (Reprinted with permission from [91], copyright 2009 American Chemical Society.)

Doping of CNT-based TCFs is generally essential for further performance improvement. For instance, without further chemical doping, CNT-based TCFs exhibit sheet resistances of 300–1000 Ω/square at around 85% transmittance.[81–84] The poor conductivity can be attributed to the complexity in CNT films. CNTs are usually doped by oxygen or oxygen functional groups, which may lead to a serious deterioration in the electrical properties,[85–88] because those covalent bonds on the CNT sidewalls cause the localization of the delocalized π electrons occupying a one-dimensional density of states. The spacing between CNTs, due to the weak van der Waals interaction, may significantly increase the overall resistance by a factor as high as 10.[89,90] Moreover, CNT films generally contain 33% metallic and 67% semiconducting CNTs. Previous reports have shown that metallic CNTs conduct well to other metallic CNTs, and semiconducting CNTs also conduct well to other semiconducting CNTs, but metallic/semiconducting CNT contacts cause a Schottky barrier, which greatly suppresses the electrical transport. Topinka et al*.* [91] investigated this phenomenon with an electronic phase diagram, as shown in Figure 3. It showed the influences of CNT type and configuration on the electrical conduction of a series of simulated CNT networks, where CNTs are varied from metallic to semiconducting in the simulation. For pure metallic or semiconducting CNTs, the current through the film is quite uniform, and the conductance is very high. However, for the film containing around 20% metallic and 80% semiconducting CNTs, no conduction can be observed.

Chemical modification or doping is a common method to enhance the electrical conductivity of CNT films. Many dopants including halogen (Br_2_),[92] acids (HNO_3_, HCl),[93,94] chlorides (FeCl_3_, SoCl_2_),[95,96] metal oxides (MoO_*x*_),[97] and organic molecules [98] have been investigated. These dopants encourage the charge transfer between them and CNT, and enable Fermi level tuning of CNT for the desired electronic properties. Moreover, doping treatments can reduce the CNT–CNT contact resistance and increase the carrier concentration of semiconducting CNT, which can provide more conductive films than metallic CNTs whether or not they are doped.[99,100] However, most dopants are not stable to air, temperature, or humidity, resulting in unstable electrical properties in CNT films. Therefore, the reliability of doping is becoming one of the biggest challenges for practical applications.

A multiple-step fabrication process, including dispersing CNTs, depositing CNT films, removing surfactants and doping CNT films, has been the most used method for CNT-based TCFs. For example, as shown in Figure 2, Shim et al*.* [101] demonstrated a typical method to fabricate CNT-based TCFs via spray-coating SDBS-aided dispersion. After removal of the SDBS, solutions of transition metal slats including AuCl_3_, IrCl_3_, Ni(NO_3_)_3_ were utilized for doping, which resulted in the best CNT-based TCFs with sheet resistance of 92 Ω/square at 83.8% transmittance. Kim et al*.* [94] used hydroxypropylcellulose (HPC), a derivative of cellulose to dispersing the CNTs. The viscosity of CNT ink can be easily controlled by adjusting the concentrations of HPC or CNTs. CNT-based TCFs with different thicknesses were deposited by a doctor-blade method. They developed a photonic curing process to remove the HPC polymer. This system used flash lamps to deliver continuously adjustable, megawatt-intensity, microsecond-resolution pulses of broad-spectrum light to achieve a rapid heating and cooling process. This process enabled a high-temperature process, while the damage from such heating treatment to low-temperature plastic substrate could be avoided. After removal of the matrix polymer, HNO_3_ doping resulted in highly conductive CNT-based TCFs with sheet resistance of 68 Ω/square at 89% transmittance. The negligible change in sheet resistance after 200,000 cycles of bending identified the superior flexibility of CNT-based TCFs.

However, the above multiple-step methods are too complicated and costly for mass production of TCFs. The choice of surfactants is crucial to simplifying the fabrication process. Some acids have been developed for dispersing CNTs, so further doping treatment is not necessary. Hecht et al*.* [102] used chlorosulfonic acid (CSA) as a dispersant. CNTs were well dispersed by just stirring the solvent mixing with 10 mg and 50 ml CSA for 12 h. Sonication, which could introduce damage along CNT walls, and dramatically shorten CNT length, was not necessary in this work. Vacuum filtration was used to collect CNT films, which were then transferred to a plastic substrate. As-prepared CNT-based TCFs exhibited a sheet resistance of 60 Ω/square at 90.9% transmittance. Moreover, Mirri et al*.* [103] demonstrated a scalable dip coating method to fabricate CNT-based TCFs. They also used CSA to disperse the single- and double- walled CNTs with high concentrations up to 3 mg ml^–1^. After film deposition, the CSA can be easily removed by coagulation or drying, followed by washing in chloroform. Without further doping, the at ∼88% transmittance, single- and double-walled films had a sheet resistance of ∼1300 and 140 Ω/square, respectively. Mirri et al*.* [103] attributed the higher performance for double-walled CNTs to the length and quality. The sheet resistances of the films are comparable with those after acid doping. However, the sheet resistances were sensitive to the air, temperature and humidity. After 24 h at 85% relative humidity and 85°C, the film sheet resistance increased by 220%. More research is needed to develop stable doping methods that satisfy the industrial standard for accelerated ageing tests.

**Figure 4.**
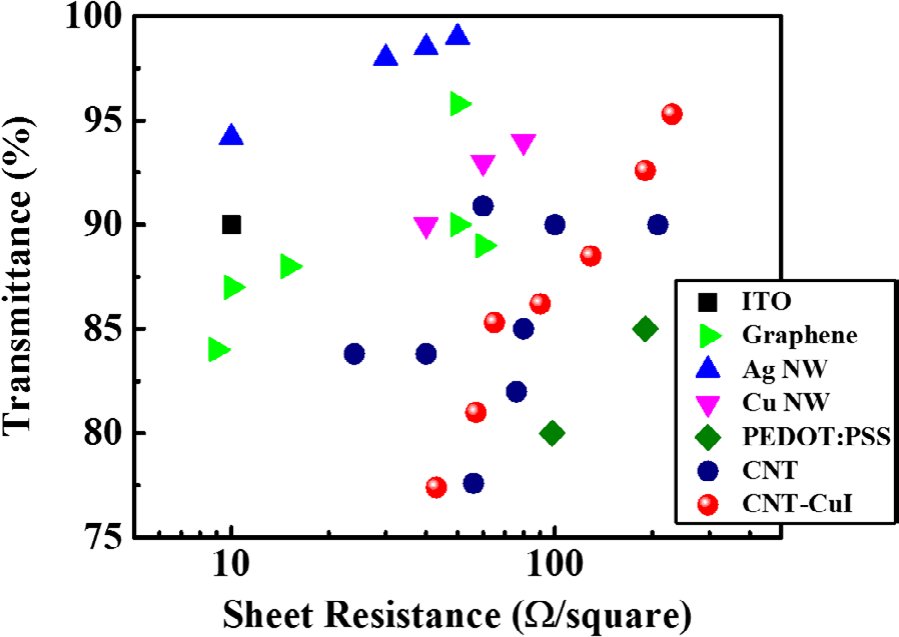
Plot of specular transmittance versus sheet resistance for the best performances of CNT-based TCFs.[96,102,104] Values for commercial ITO, the best TCFs using graphene,[28–31] Ag NW,[19] Cu NW [18] and PEDOT:PSS [12] are provided for comparison.

We summarize the best CNT-based TCFs in Table 1. The performances of some CNT-based TCFs are promising for the replacement of ITO, but other ITO alternatives such as metal nanowires and graphene exhibit better performance. Figure 4 shows the best transmittance and sheet resistance reported in the literature to date for CNTs, graphene and metal nanowires. Significant progress is still required to improve the performances of CNT-based TCFs. However, constructing CNT hybrid composites with graphene and/or metal nanowires may lead to a breakthrough over the conductivity limits in CNT-based TCFs.[104–115]

**Table 1.** Summary of the best CNT-based TCFs. PET stands for polyethylene terephthalate, and NMP for N-methyl-2-pyrrolidone.

Substrate	Dispersant	Coating	Doping	*R*_*s*_ (Ω/sq)	T. (%)	Ref.
PET	Superacid	Filtration	None	60	90.9	102
Glass	Superacid	Dip coating	None	140	88	103
PET	SDS	Spin coating	HNO_3_	80	85	39
PET	HPC	Doctor blade	HNO_3_	68	89	96
Glass	Oleum	Filtration	HNO_3_	76	82	84
Quartz	Triton X-100	Filtration	SoCl_2_	56	78	95
Glass	NMP	Spray coating	MoO_3_	100	85	97
Glass	HPC	Doctor blade	CuI	65	85	104
PET	SDS	Filtration	Silver nanowire	26	90	105

## 3. Stability of CNT-based TCFs

The chemical bonding of nanotubes is composed entirely of sp^2^ bonds, similar to graphite. These bonds are stronger than the sp^3^ bonds in diamond, and accordingly provide CNT with outstanding strength. The thermal stability of CNTs is estimated to be up to 2800°C in a vacuum and around 750°C in air.[116] However, it is quite different in the case of CNT-based TCFs. Doping is usually essential for further performance improvement. The conductivity of CNT films can be greatly enhanced through chemically doping treatment, which results in the enhancement of charge carrier and decrease of contact resistance of CNT–CNT junction. However, doped CNT films become sensitive to air, temperature, or humidity. Thus, most of the reported properties of CNT-based TCFs are not stable.

### 3.1. General doping

**Figure 5.**
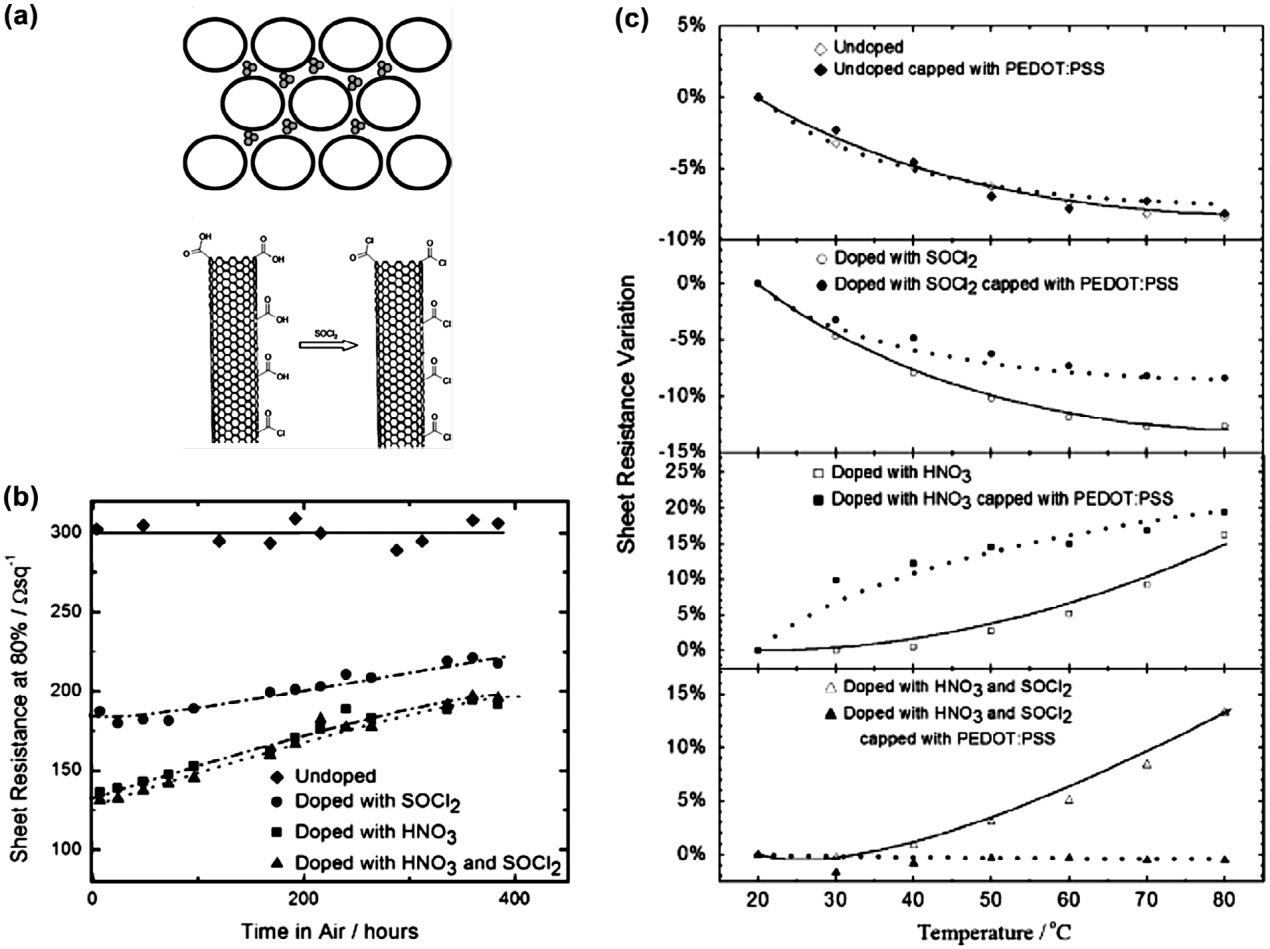
(a) (top) Simple space filling model of intercalated HNO_3_ molecules within a CNT unit, and (bottom) nucleophilic substitution of carboxylic acid groups on the sidewalls and tips of the CNT with acyl chlorides via chemical treatment with SOCl_2_. (b) Absolute sheet resistance versus time in air of four CNT-based TCFs. (c) Sheet resistance increases with temperature for undoped, SOCl_2_-, HNO_3_- doped CNT-based TCFs. (Reprinted with permission from [117], copyright 2008 Wiley-VCH.)

As efficient p-type dopants, strong acids and inorganic solvent such as HNO_3_ and/or SOCl_2_ have been widely used to fabricate CNT-based TCFs with high conductivity. Jackson et al*.* [117] systematically investigated the stability of undoped and doped CNT-based TCFs. As shown in the top of Figure 5(a), HNO_3_ molecules and/or NO_*x*_ residues dope the CNTs by intercalating the CNTs within the network. The doping effects of HNO_3_ have been shown to be easily reversible, resulting in a quick increase in sheet resistance of doped CNT films. On the other hand, SOCl_2_ doping leads to the nucleophilic substitution of carboxylic acid groups on the sidewalls and tips of the CNT with more electronegative acyl chlorides. Although the acyl chlorides groups are covalently bonded to CNTs, the bonding structures are known to be very reactive, resulting in a poor overall stability. The best results for CNT-based TCFs using strong-acid doping are not stable. The stability problem could be more severe during the actual device process, which may contain chemical treatment, solution deposition, vacuum evaporation, and/or high-temperature annealing. Figure 5(b) shows the evolution of the stability of undoped and doped CNT-based TCFs upon exposure to air atmosphere. The sheet resistance of the undoped film was stable at 300 Ω/square over 400 h, while doped films showed a significant increase in the sheet resistance. The percentage increase in the sheet resistance of HNO_3_-doped film was larger than that of SOCl_2_ doped films, while the sheet resistance of HNO_3_-doped film was still lower after 400 h. Moreover, they also investigated the effects of heating on the doping stability, as shown in Figure 5(c). The sheet resistances of undoped CNT films with or without PEDOT/PSS decreased slightly with increasing temperature. The CNT films treated with SOCl_2_ also showed a temperature dependent electrical resistance which decreased by up to 10% with increasing temperatures from 20 to 80°C. However, the CNT films treated with HNO_3_ demonstrated an increase in resistance with increasing temperature. The HNO_3_ treated film with a layer of PEDOT:PSS has an initial sharp increase in resistance with increasing temperature in comparison to the HNO_3_ doped film without PEDOT:PSS. This could be explained by the interaction of the HNO_3_ molecules on the surface of the CNT film with the PEDOT:PSS layer. The films doped with a combination of HNO_3_ plus SOCl_2_ showed no change in sheet resistance upon heating up to 80°C when the film was capped with PEDOT:PSS, while the resistance of uncapped doped films increased by more than 13% at elevated temperatures. Thus, the capping layer showed the ability to stabilize the temperature dependence of the sheet resistance.

### 3.2. Doping with MoO_*x*_

**Figure 6.**
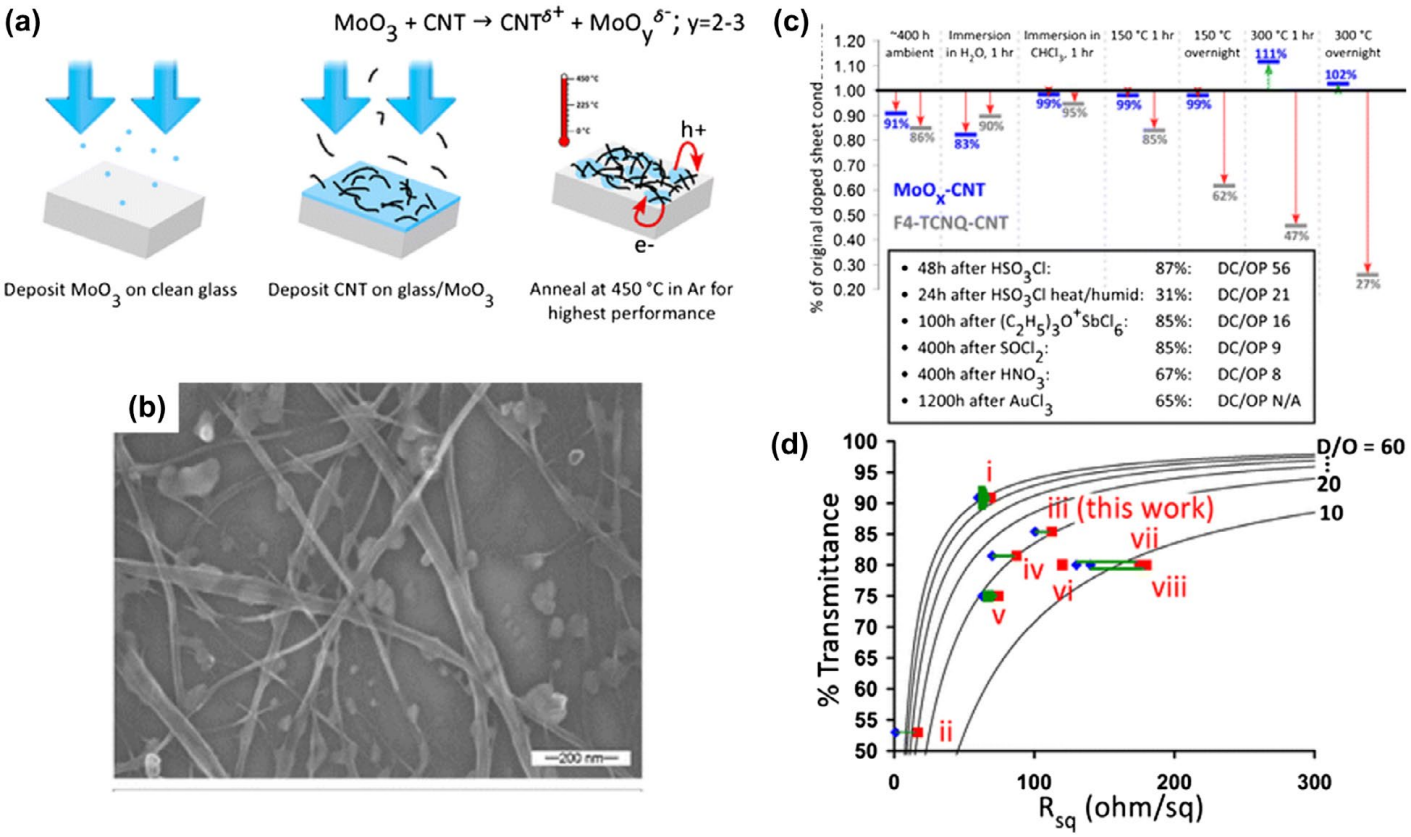
(a) Schematic showing the fabrication of CNT-MoO_*x*_ TCFs. (b) SEM micrograph of an annealed CNT-MoO_*x*_ composite film. (c) Variations of MoO_*x*_- and F4-TCNQ-doped CNT films to different thermal and chemical stressors. (d) Summary showing sheet resistances and transmittances of TCFs. (Reprinted with permission from [97], copyright 2012 American Chemical Society.)

Development of stable and reliable doping is therefore becoming the biggest challenge for practical application of CNT-based TCFs. In 2012, Hellstrom et al*.* [97] reported an interesting charge-transfer interaction between CNT networks and MoO_*x*_, and developed a stable p-type doped CNT-MoO_*x*_ bilayer TCFs. Figure 6(a) illustrates the basic process for fabricating CNT-MoO_*x*_ hybrid films. MoO_*x*_ films with a thickness of less than 10 nm were essentially transparent in the visible as initially deposited by vacuum evaporation. After annealing at 450–500°C in Ar, the CNT-MoO_*x*_ hybrid films exhibited a sheet resistance of 100 Ω/square at 85% transmittance. Further depositing a PEDOT:PSS layer resulted in sheet resistances of 80 Ω/square at 83% transmittance. Scanning electron microscopy images showed that after annealing the MoO_*x*_ layer dewetted from glass supporting substrates and did not remain continuous. This indicated that MoO_*x*_ nanoparticles, rather than continuous film, enhanced the charge transfer doping, which led to a great improvement in the electrical conductivity. Interestingly, CNT-MoO_*x*_ hybrid films exhibited good thermal stability. Figure 6(c) gives the relative variations of various stressors on the sheet resistance of CNT-MoO_*x*_ hybrid films, compared with similar data on CNT films doped with 2,3,5,6-tetrafluoro-7,7,8,8-tetracyanoquinodimethane (F4-TCNQ), and to alternative dopants in the literatures. It seems that MoO_*x*_ was the most stable of the strong CNT dopants. In ambient conditions over 20 days, sheet resistances changed slightly. Apparently, CNT-MoO_*x*_ hybrids had good chemical stability over F4-TCNQ doped samples subject to every chemical test performed except for 1 h immersion in water. This instability is most likely due to the solubility of MoO_*x*_ in water. This is an interesting work using nanoparticles of semiconductor rather than thin film or metallic nanoparticles to achieve efficient electrical transport in CNT networks. The excellent stability makes CNT-MoO_*x*_ hybrids extremely attractive candidates for practical transparent electrodes.

### 3.3. Doping with copper halides

**Figure 7.**
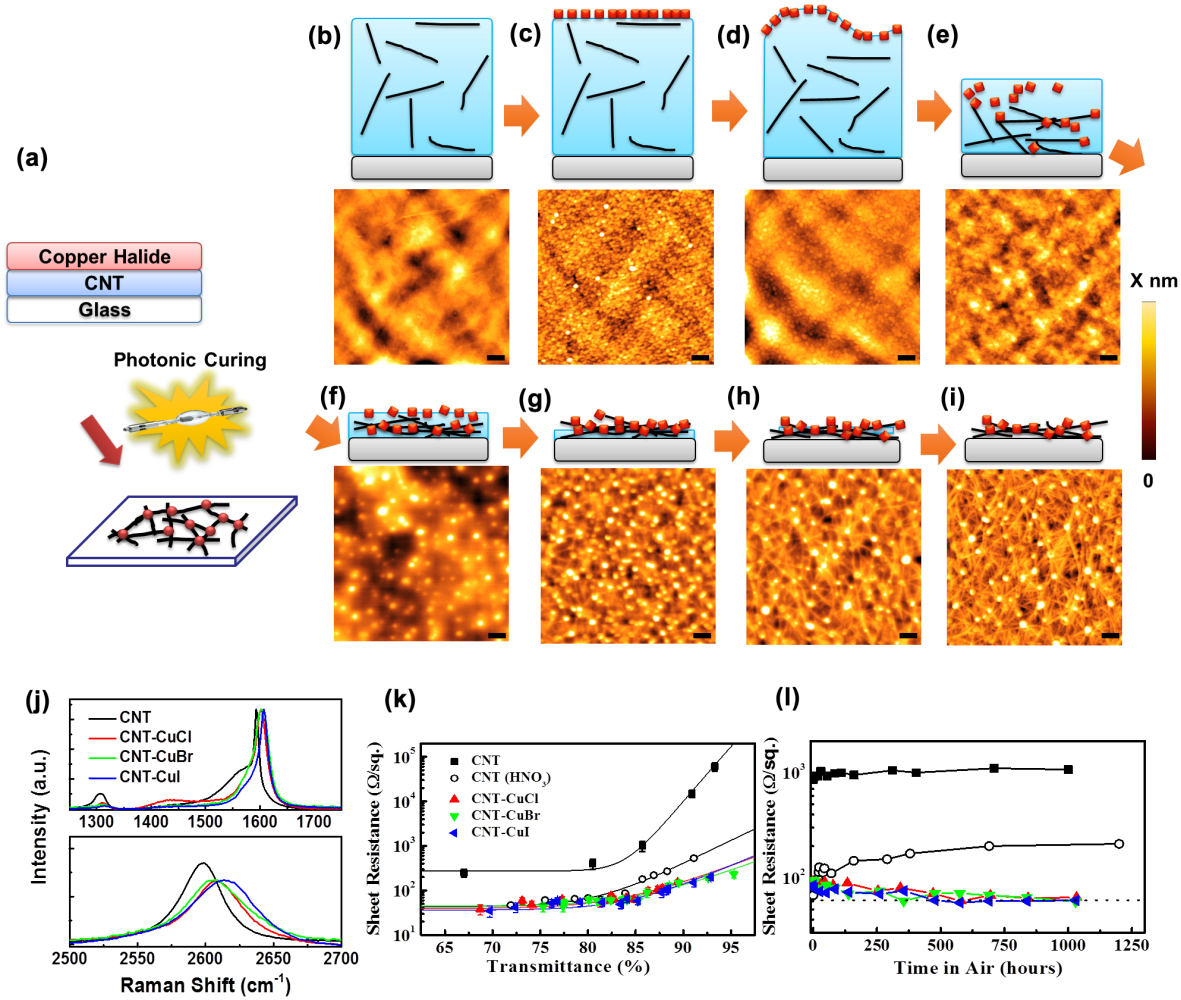
(a) Schematic showing the photonic curing process for fabricating CNT-copper halide hybrids. Formation mechanism of the interconnecting nodes, and corresponding atomic force microscopy (AFM) images of (b) as-deposited HPC-dispersed CNT, (c–j) as-deposited CNT-HPC/CuI films during the photonic curing. Raman spectra of (k) CNT and CNT-copper halide films after photonic curing. Properties of CNT-copper halide films as transparent electrodes: (i) sheet resistance versus transmittance, (m) durability of sheet resistance in air at room temperature. (Reprinted with permission from [104], copyright 2015 Elsevier.)

Recently, we have demonstrated a doping technology to build interconnecting nodes in CNT networks with copper-halide crystallites.[104] The technique is schematically illustrated in Figure 7(a). Thin films of copper halides can be prepared by either vacuum- or solution-based processes. A pulse photonic curing system was used to produce a rapid heating and cooling process at a microsecond timescale. This rapid heating and cooling process enabled the manipulation of copper-halide crystallites, which not only resulted in the formation of CNT-CNT interconnecting nodes, but also improved charge transfer doping. Figure 7(b–j) shows the formation mechanism, and their corresponding atomic force microscopy (AFM) images. As-evaporated CuI film exhibited polycrystalline geometry with sphere-like grains. The photonic curing process with optimized parameters was developed to remove polymer dispersant and construct halide nodes. These CuI crystallites were individually located at the cross points of two or more CNT bundles. Besides the novel structure, doping with copper halides significantly decreased the intensity of D-band signals. The blueshifts indicated p-type doping, where charges transferred from CNTs to halides occurred, corresponding to a downshift of Fermi level toward the valence band of CNT. The CuI-HPC/halide films exhibited an initial sheet resistance of over 10^6^ Ω/square, while the photonic curing led to a remarkable improvement in their electrical conductivity. With introduction of halide nodes, the CNT films exhibited 90–110 and 55–65 Ω/square at 90% and 85% transmittance, respectively. Similar results were obtained for the CNT films containing CuCl, CuBr or CuI. On the other hand, un-doped CNT films exhibited stable sheet resistances. After HNO_3_ doping, CNT film exhibited an initial sheet resistance of 65 Ω/square at 83% transmittance, and it was rapidly increased to 130 Ω/square after 24 h exposure to air. The CNT-halide films showed extremely stable sheet resistance values after air exposure for more than 1000 h at room temperature.

**Figure 8.**
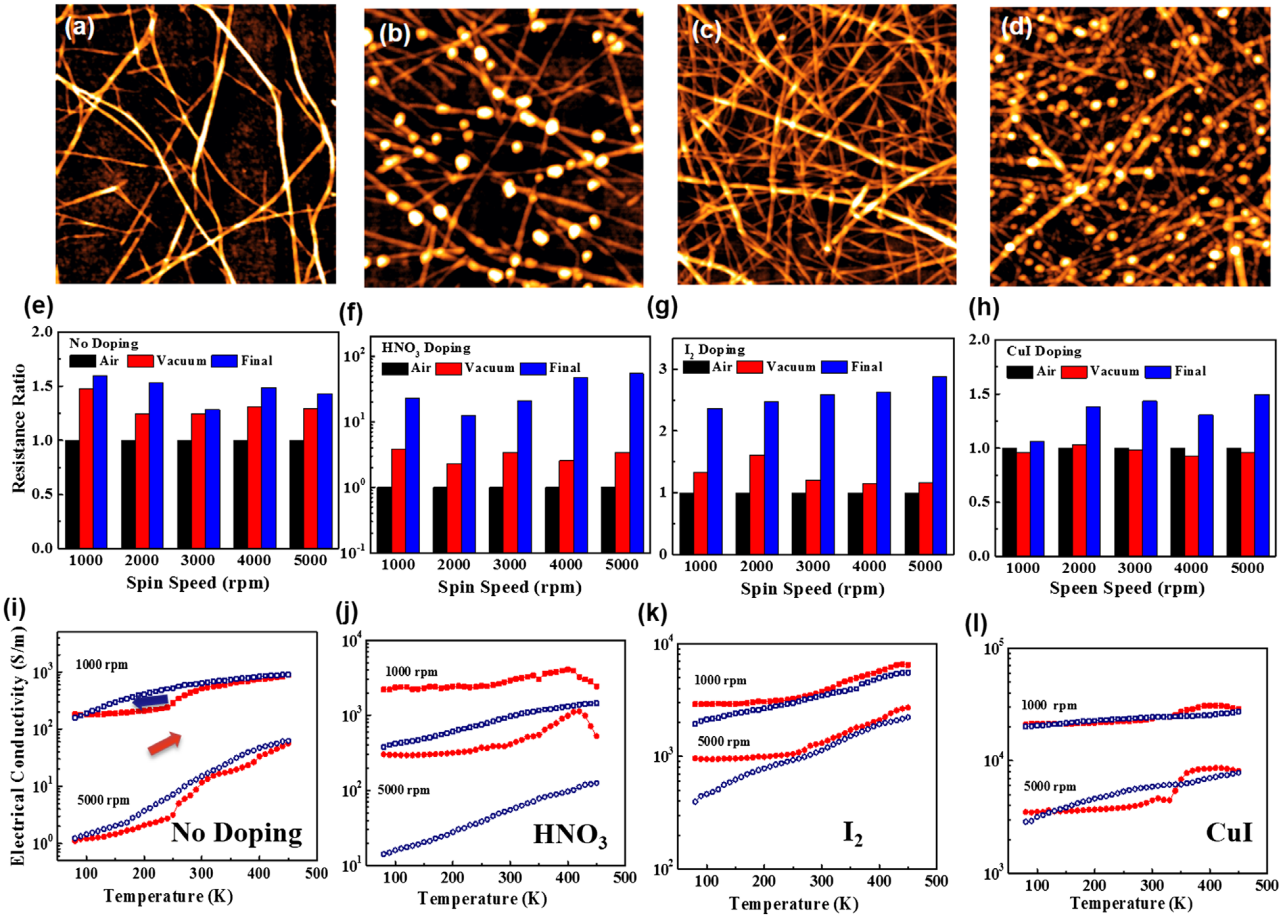
AFM images of CNT films spin-coated with spin speeds of (a) 5000 rpm, and (c) 1000 rpm and their corresponding CNT-CuI hybrid films (b) and (d). The resistance variations of CNT networks (e–h) and the temperature dependent conductivity (i–l) of CNT films with different doping treatments. (Reprinted with permission from [118], copyright 2015 American Institute of Physics.)

In order to understand such technology, we have further investigated the effects of different doping methods including HNO_3_ solution, I_2_ vapor and CuI solid.[118] Figure 8 shows the surface morphology of the undoped CNT films. CuI doping led to a unique CNT-CuI hybrid film. The effects of environments such as vacuuming or heating were also investigated. For undoped films, the resistances increased by a factor of 1.2–1.4 after vacuuming, and further increased after heating. Vacuuming and heating can remove the weakly absorbed dopant molecules. Desorption of the gaseous molecules could be the reason for the increased resistance in undoped films by vacuuming. For HNO_3_ doping, vacuuming processes led to a threefold increase in resistance. Further, a more than fivefold increase induced by heating suggested that CNT films lose almost all of the HNO_3_ induced dopant molecules. CNT-CuI hybrid films exhibited extremely stable electrical performances; the resistance was only increased by factors in a range of 1.1–1.5. The detailed temperature dependence was also investigated. The contact resistance rather than the resistance of individual CNT was more dominant in thinner films. The contact resistances decreased with temperature, because carriers crossed the potential barrier between CNTs more easily at higher temperature. Therefore, thinner undoped films seem to be more semiconducting. Moreover, a hysteresis loop was observed in the forward-reverse temperature cycle. This may be attributed to the variations in contact resistance due to desorption of molecules intercalated in CNT bundles at high temperature. Doping suppressed the potential barrier for carrier transfer between CNTs, and, consequently, doped CNT films became more metallic. Higher temperature activated the ionic dopants intercalating the CNT bundles, and simultaneously removed the volatile dopants from CNTs. Overall, CNT-CuI hybrid films exhibited the smallest hysteresis, demonstrating their excellent stability.

These results suggest that semiconductor nanoparticles can be promising dopants, and can be used to form a stable hybrid composite with CNTs, but do not degrade the electronic and transport properties of CNTs. In particular, the processing feasibility and excellent reliability indicate a good outlook for CNT-CuI hybrid films in practical applications. On the other hand, both CuI and MoO_*x*_ are typical wide-bandgap semiconductors with deep work function of above 5.2 eV, being close to the highest occupied molecular orbital (HOMO) of organic donor molecules. MoO_*x*_ is also used as an electron acceptor for doping organic semiconductor and CNTs, while CuI can act a structural template to control the molecular growth to improve the OPV cells.[119–121] Those strong and stable doping technologies may push the application of CNT-based TCFs in OPV devices.

## 4. Application of CNT-based TCFs

TCFs are a widely used technology in daily life for various optoelectronics. Although ITO is dominating the current market in practical applications of TCFs, more and more commercial products using ITO alternatives including CNTs, metal mesh as well as metal nanowire are appearing. In this section, we review recent progress, and discuss the challenges in sensing devices (touch panel), OPV and OLED devices which use CNT-based TCFs.

### 4.1. Sensing devices

Touch panels, i.e. input devices on the top of electronic displays, are the most widely used sensing devices. Touch panels have been widely used as a direct human/device communication tool in various digital devices such as tablets and smart phones, and demand for them is rapidly growing. Touch panels can be resistive-type or capacitive-type. A resistive touch panel contains several layers, the most important of which are two layers of TCFs separated by a thin space, while a capacitive touch panel contains an insulator and a layer of TCF.[122] Resistive touch panels usually need TCF with higher transmittance (>95%), while capacitive touch panels need TCF with smaller sheet resistance (<300 Ω/square). Touch panels have no special requirements for surface roughness or the work function of TCFs, and the requirement for sheet resistance is not strict. Touch panels may be one of the most promising practical applications for CNTs-based TCFs.[122–128] However, other low-cost technologies including metal mesh and metal nanowires are also receiving increased attention. Therefore, besides the performances of transmittance/sheet resistance, the price is becoming a crucial factor for the choice of TCFs.

In 2013, Foxconn Electronics (Hon Hai Precision Industry, China) announced that they developed a technology (CNTouch) for manufacturing touch panels. This technology, invented by Feng [44], uses a straightforward drawing process to directly transfer the vertical alignment of double walled CNTs in 200 mm silicon wafer into a substrate. CNTouch can provide a low-cost process for mass production of touch panels using CNT-based TCFs. Currently, the Tianjin production lines of CNTouch are able to produce three million 102 mm panels per month and its production lines in Guiyang, China are currently able to deliver about three million 127 mm panels per month. More and more companies are considering these low-cost touch panels, and some products such as Huawei (Shenzhen, China) and HTC (Taiwan, China) smartphones may have already used these touch panels. Also in 2013, Canatu developed a CNB technology to manufacture touch panels in Finland. The key benefits of CNB TCFs are high flexibility, cost competitiveness, and optical transmission. Interestingly, both Foxconn and Canatu use dry processes to manufacture CNT-based TCFs. Although these technologies use very special CNTs, very low-cost processes have been developed for mass production of CNT-based TCFs, which may lead to them being competitive among other TCFs.

**Figure 9.**
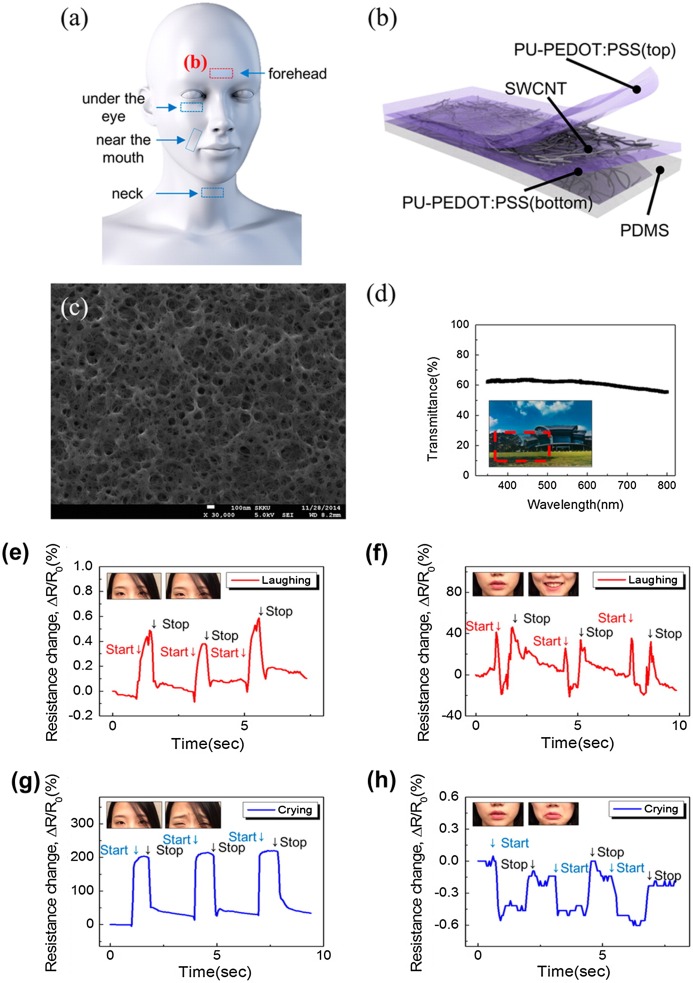
(a) Schematic illustration of stretchable transparent strain sensors. (b) Schematic illustration of the strain sensor. SWCNT stands for single-walled CNT, PDMS for polydimethylsiloxane and PU for polyurethane. (c) Top-view FE-SEM image and (d) transmittance spectra of the three-layer stacked sensor. Time-dependent responses of the sensor attached to the (e) forehead and (f) skin near the mouth when the subject was laughing and of the sensor attached on the (g) forehead and (h) skin near the mouth when the subject was crying. (Reprinted with permission from [144], copyright 2015 American Chemical Society.)

As well as the application for touch panels, various sensing devices using CNT-based TCFs have been attracting special interest as wearable electronics. Since the electrical properties of CNT networks can be very sensitive to temperature, humidity, light, gas, pressure and strain, they can transfer the variation of the environment into electronic signals.[129–143] In particular, they can describe abstract experiences such as human action, feeling and emotion with digital signals. In 2015, Roh et al*.* [144] reported a stretchable, transparent strain sensor for human–machine interfaces comprising a hybrid of CNT and conductive elastomers, as shown in Figure 9. The percolating networks between conductive CNTs and PEDOT improved the tunability of strain sensitivity, stability, and optical transparency. Such wet-processed sensors exhibited enough sensitivity to detect small strains induced by emotional expressions such as laughing and crying, as well as eye movement. The sensor can become an interface between humans and robots, and can help machines read human feeling and emotion. The rapid development of robots and artificial intelligence will further motivate studies on sensing devices used CNT-based TCFs.

### 4.2. OPV devices

OPV cells including hybrid perovskite photovoltaics have attracted much interest in both academic and industrial fields, due to their potential as a promising source of renewable energy. In recent decades, significant progress in OPV cells has been achieved by the development of molecules and device fabrication processes. However, those works have mostly been carried out on ITO coated glass substrates. The brittle ITO film is not applicable for future OPV cells, which should be flexible on plastic substrates in order to differentiate them from conventional crystalline Si PV cells. Therefore, establishing the fabrication process on flexible electrodes is of great importance for boosting OPV cells for daily use.

As shown in Table 1, even the best CNT-based TCFs exhibit a sheet resistance of 60 Ω/square at 90.9% transmittance. Considering that commercial ITO exhibits a sheet resistance of 10 Ω/square at 90.9% transmittance, such high sheet resistance is not acceptable for efficient OPV cells. CNT-based TCFs have been widely investigated for OPV applications.[145–165] Table 2 summarizes the best OPVs fabricated on CNT-based TCFs, and the best OPV cells on ITO-free TCFs. Typically, PEDOT:PSS is deposited on CNT-based TCFs as a hole transport layer. Because the rough CNTs exhibit hydrophobic characteristics, a PEDOT:PSS coating can smoothen the surface and improve the wettability of CNT films. Moreover, high-conductivity PEDOT:PSS (such as Clevios PH 1000) is often introduced to improve the conductivity of CNT-based TCFs. Therefore, most of the reported OPV device structures are substrate/CNT (without high-conductivity PEDOT:PSS)-based TCFs /PEDOT:PSS/polymer: phenyl-C_61_-butyric-acid-methyl ester (PCBM)/Al, where polymer includes poly(3-hexylthiophene-2,5-diyl) (P3HT) or poly({4,8-bis[(2-ethylhexyl)oxy]benzo[1,2-b:4,5-b′]dithiophene-2,6-diyl}{3-fluoro-2-[(2-ethylhexyl)carbonyl]thieno[3,4-b]thiophenediyl}) (PTB7), and LiF or TiO_2_ is used as an electron extraction layer. The substrate can use transparent plastic materials (PEN:　polyethylene naphthalate) to realize flexible and/or stretchable OPV cells. The high sheet resistance of CNT-based TCFs usually leads to a degradation in overall efficiency of OPV cells, compared with the reference cells on ITO substrate. In particular, acid-doped CNT-based TCFs exhibit unstable sheet resistances, which will degrade during the device process. Note that the best OPVs usually used thick CNT films in order to achieve smaller sheet resistance. The results imply that a sheet resistance less than 50 Ω/square is required for efficient OPV cells with a high fill factor (FF).

**Table 2.** Summary of the best OPV cells on CNT-based TCFs and other ITO-free TCFs. BCP stands for bathocuproine, GO for graphene oxide and PEI for polythyleneimine.

Device structure	CNT-Based TCFs method + doping	*R*_*s*_ (Ω/sq)	T. (%)	Area (mm^2^)	PCE (%)	Ref.
Glass/CNT/PEDOT:PSS/P3HT:PCBM/Ca/Al	Spray + acid	60	70	3	3.1	145
Glass/CNT/PEDOT:PSS/P3HT:PCBM/LiF/Al	Spray + acid	128	90	N/A	2.3	146
Glass/CNT/PEDOT:PSS/P3HT:PCBM/Ca/Al	Spray + acid	56	65	4	4.1	147
Glass/CNT/PEDOT:PSS/P3HT:PCBM/LiF/Al	Dip + acid	188	92	4	2.0	148
Glass/CNT/PEDOT:PSS/P3HT:PCBM/LiF/Al	Filtration + MoO_3_	326	90	9	2.4	152
Glass/CNT/PEDOT:PSS/PTB7:PCBM/LiF/Al	Filtration + MoO_3_	84	65	9	6.0	152
PET/CNT/PEDOT:PSS/PTB7:PCBM/LiF/Al	Filtration + MoO_3_	84	65	9	3.9	152
PET/PANI:CNT/PEDOT:PSS/F82T/C_60_/Al	Dip + meta-cresol	295	89	N/A	2.3	149
PET/CNT/PEDOT:PSS/ P3HT:PCBM/Al	Filtration + none	200	85	4	2.5	150
Glass/CNT:PEDOT:PSS/PEIE/ZnO/PBDTTT-CT:PCBM/V_2_O_5_-GO/Ag	Spin +PEDOT:PSS	40	85	19.7	7.5	154
Glass/CNT/PEDOT:PSS/DIP/DBP/C_60_/BCP/Al	Blade + CuI	70	80	6	3.0	153
Glass/CNT/PEDOT:PSS/perovskite/PCBM/Al	Filtration + acid	25	70	9	6.3	167
PET/CNT/PEDOT:PSS/perovskite/PCBM/Al	Filtration + acid	25	70	9	5.4	167
FTO/TiO_2_/perovskite/spiro/CNT	Lamination + none	2000	60	16	6.3	168
Glass/CuNW/PEDOT:PSS/F_4_ZnPc:C_60_/BPhen/Al	Spray	25	83	16	3.1	169
Glass/AgNW:PEDOT:PSS/PEDOT:PSS/polymer (not commercial):PC_71_BM/TiO_2_/Al	Rod	8	92	15	7.4	170
PEN/PEI/Ag/PEDOT:PSS/PEDOT:PSS/PEI:PTB7-Th:PC_70_BM/MoOx/Ag	-	9	90	4.6	9.8	172
PET/Ag mesh:PEDOT:PSS/ PEDOT:PSS/perovskite/PCBM/Al	Nano-imprinting	3	85	10	14.0	173

**Figure 10.**
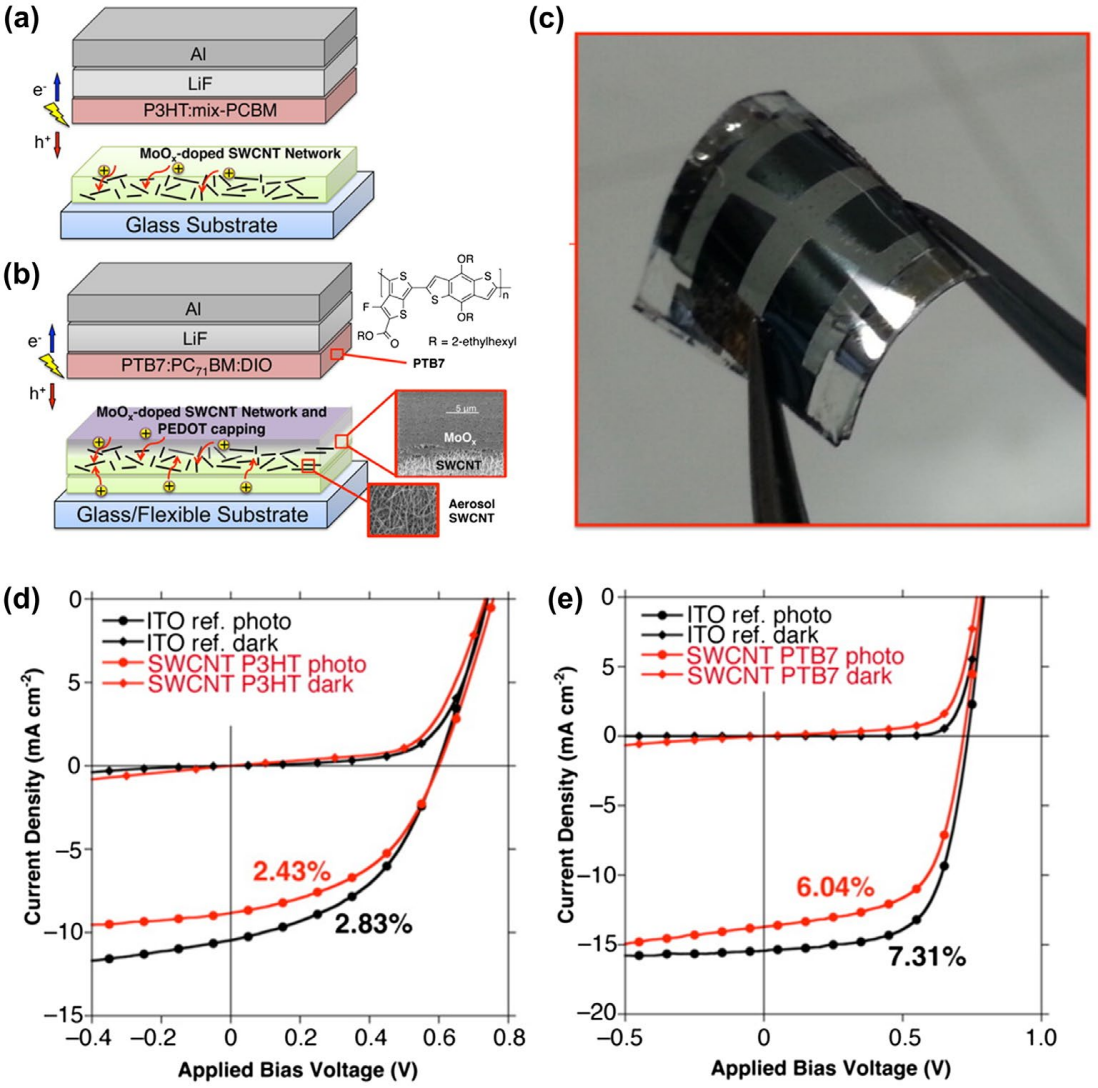
(a) P3HT-based cells and (b) the most optimized PTB7-based device. (c) Photograph of the flexible OPV on CNT-based TCFs. *J*–*V* curves of the: (d) P3HT:mix-PBM-based devices and (e) PTB7:PC_71_BM-based devices. (Reprinted with permission from [152], copyright 2015 American Chemical Society.)

Stable dopants are of significance for CNT-based TCFs in OPV applications. MoO_*x*_ and CuI could be promising candidates. In 2015, Jeon et al. [152] fabricated a series of OPV cells on CNT-based TCFs, as shown in Figure 10. They used dry-processed CNT-based TCFs, which was sandwich-doped with two layers of MoO_*x*_. As a result, the sheet resistance became 84 Ω/square at 65% transmittance. Doping with MoO_*x*_ not only greatly improved the conductivity of CNT films, but also modified the work function of CNTs for efficient hole extraction. Thus, the CNT-MoO_*x*_ films were regarded as electron-blocking transparent electrodes. The OPV cells showed power conversion efficiencies (PCE) of 2.4% and 6.0% using P3HT:PCBM and PTB7:PCBM as active layers, respectively. Although these PCE values were 80% of those for ITO-based OPV cells, it was the most efficient OPV cells on CNT-based TCFs in literature to date. When PET substrate was used, the OPV cell exhibited a PCE of 3.9%, which was capable of withstanding a severe cyclic flex test. It showed that stable CNT-based TCFs can be used for polymer OPV cells with high efficiency, being comparable to ITO devices.

**Figure 11.**
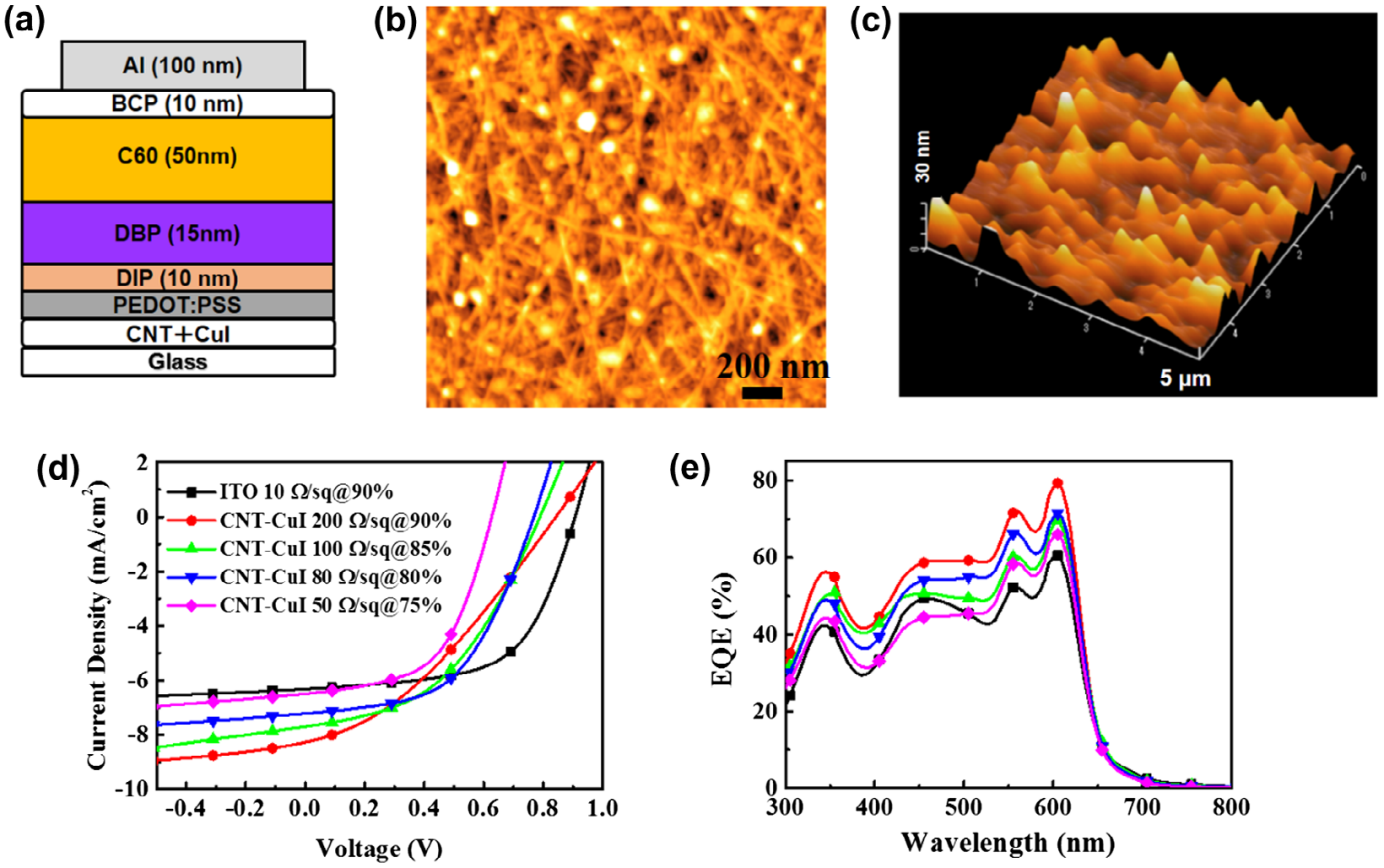
(a) Device structure, AFM images of the CNT-CuI hybrid (b) before and (c) after deposition of PEDOT:PSS film. (d) *J*–*V* characteristics under 1-sun illumination and (e) EQE of the OPV cells on CNT-CuI. (Reprinted with permission from [153], copyright 2016 Royal Society of Chemistry.)

On the other hand, the stability problem could be more severe during actual device process, especially in vacuum process. Very few papers report the vacuum-evaporated OPV cells using CNT as transparent electrodes. We developed a full solution process to fabricate CNT-CuI hybrid films by utilizing a photonic curing process.[153] OPV cells were fabricated with using tetraphenyldibenzoperiflanthene (DBP) as a donor and fullerene (C_60_) as an acceptor. Diindenoperylene (DIP) was introduced to construct a cascade-type cell architecture.[166] OPV cells on a series of CNT-CuI films were fabricated to understand the effects of transmittance and the resistance on performances in detail, as shown in Figure 11. Comparing to a reference cell fabricated on ITO, all of the cells on CNT-CuI exhibited smaller open-circuit voltage (*V*_*OC*_), and FF, but larger short-circuit current (*J*_*SC*_). Large leakage current and high sheet resistance of CNT-CuI electrode were the possible reasons. The increase in the external quantum efficiency (EQE) spectra, from 400 to 650 nm, identified that cascade structure was successfully established on CNT-CuI, and the DBP/C_60_ contact area was larger on CNT-CuI film. The OPV cells on series of CNT-CuI films exhibited very similar IQE spectra in whole wavelength, indicating that the sheet resistance of CNT-CuI played a negligible role in the efficiencies in exciton dissociation, charge transfer and charge collection. As a result, the best cell fabricated on the CNT-CuI film with 80% transmittance exhibited *J*_*SC*_ of 7.3 mA cm^–2^, *V*_*OC*_ of 0.77 V, FF of 0.53, leading to a PCE of 3.0%, being close to the cell (3.4%) on conventional ITO. It was the first efficient OPV cell based evaporating small molecules on CNT-based TCFs, which was comparable to other OPV cells using metal nanowires or graphene as TCFs. Overall, constructing a nanostructured template is an efficient strategy for improving the OPV cells whether or not ITO is used. In particular, by capitalizing on the structural characteristics (one-dimensional nanostructures) of non-traditional electrodes including CNT, metal nanowires may provide a new strategy for efficient OPV cells.

Beside the OPV cells, lead-based perovskite solar cells can be fabricated on CNT-based TCFs.[167,168] Apparently, for the efficiency game in OPV applications, CNT-based TCFs are not promising.[169–173] In particular, the flexible organic/inorganic perovskite cells exhibited a PCE of 14% on hybrid TCFs with metal mesh-PEDOT:PSS, which had a very small sheet resistance of 3 Ω/square at 85% transmittance.[173] It is necessary to further improve CNT-based TCFs with sheet resistances of below 50 Ω/square at transmittance of above 90%. Constructing hybrid structures with CNT and metal nanowires is a promising technology. Moreover, in order to achieve the required flexibility and stretchability, vacuum-evaporated metal electrodes should be also replaced with flexible electrodes. The issues of low-cost fabrication process and device stability should be addressed. Overall, it remains a large space for realizing the actual OPV application on CNT-based TCFs.

### 4.3. OLED devices

OLED is attracting more and more interest in solid-state lighting devices and digital displays in devices such as television screens, computer monitors and various portable devices. OLED is a light-emitting diode, where the emissive electroluminescent layer is an organic film that converts electrical current to light. This organic film is sandwiched with two electrodes, at least one of which is transparent.

**Figure 12.**
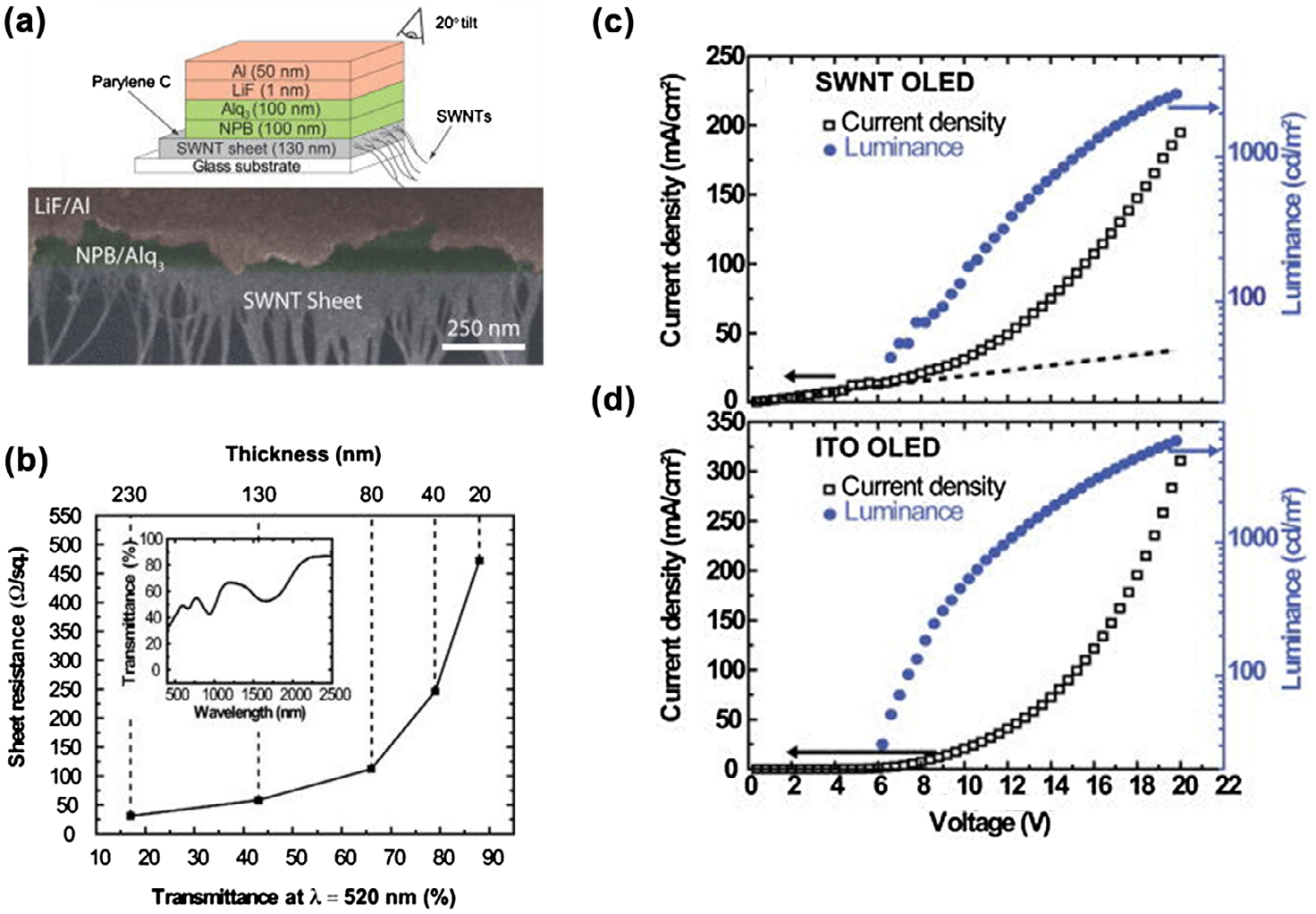
(a) Schematic of the OLED device on CNT-based electrode and corresponding cross sectional scanning electron microscopy image. (b) Sheet resistance as a function of the optical transmittance. Current density and luminance as a function of applied voltage for OLEDs fabricated on (c) CNT and (d) ITO anodes. (Reprinted with permission from [175], copyright 2006 American Institute of Physics.)

CNT-based TCFs have been widely investigated for flexible OLED devices.[82,174–194] Generally, the requirements of sheet resistance and transmittance for OLED devices is less strict than in OPV devices. Similar to OPV, there are two main OLED devices using either small molecules or polymers. Multiple-layer architecture including hole and electron injection is usually used to improve the device performance and operation lifetimes. Here, PEDOT:PSS is usually used to modify the surface of CNT-based electrodes. In 2006, three groups [82,174,175] reported small-molecule and polymer OLED devices fabricated on CNT-based electrodes, respectively. Figure 12(a) shows a schematic of a small-molecule OLED device.[175] They deposited CNT films by typical filtration and transferring processes. CNT films with a thickness of 130 nm exhibited a sheet resistance of 60 Ω/square at 44% transmittance. The organic stack structure was optimized for maximum luminance efficiency and consisted in 10-nm thick copper phthalocyanine (CuPc) as a hole injection buffer layer (HIL), 50-nm thick N,N′-bis-(1-naphthyl)-N,N′-diphenyl-1,1-biphenyl-4,4′-diamine (NPB) as a hole transport layer (HTL), and 50-nm thick tris-(8-hydroxyquinoline) aluminum (Alq_3_) as an electron transport and emissive layer. The maximum achieved brightness is 6000 cd m^–2^ for the ITO-OLED compared to roughly half, 2800 cd m^–2^, for the CNT-OLED. Considering that CNT film exhibited a low transmittance of 44%, the CNT-OLED device had similar emission performances with ITO-OLED device.

**Figure 13.**
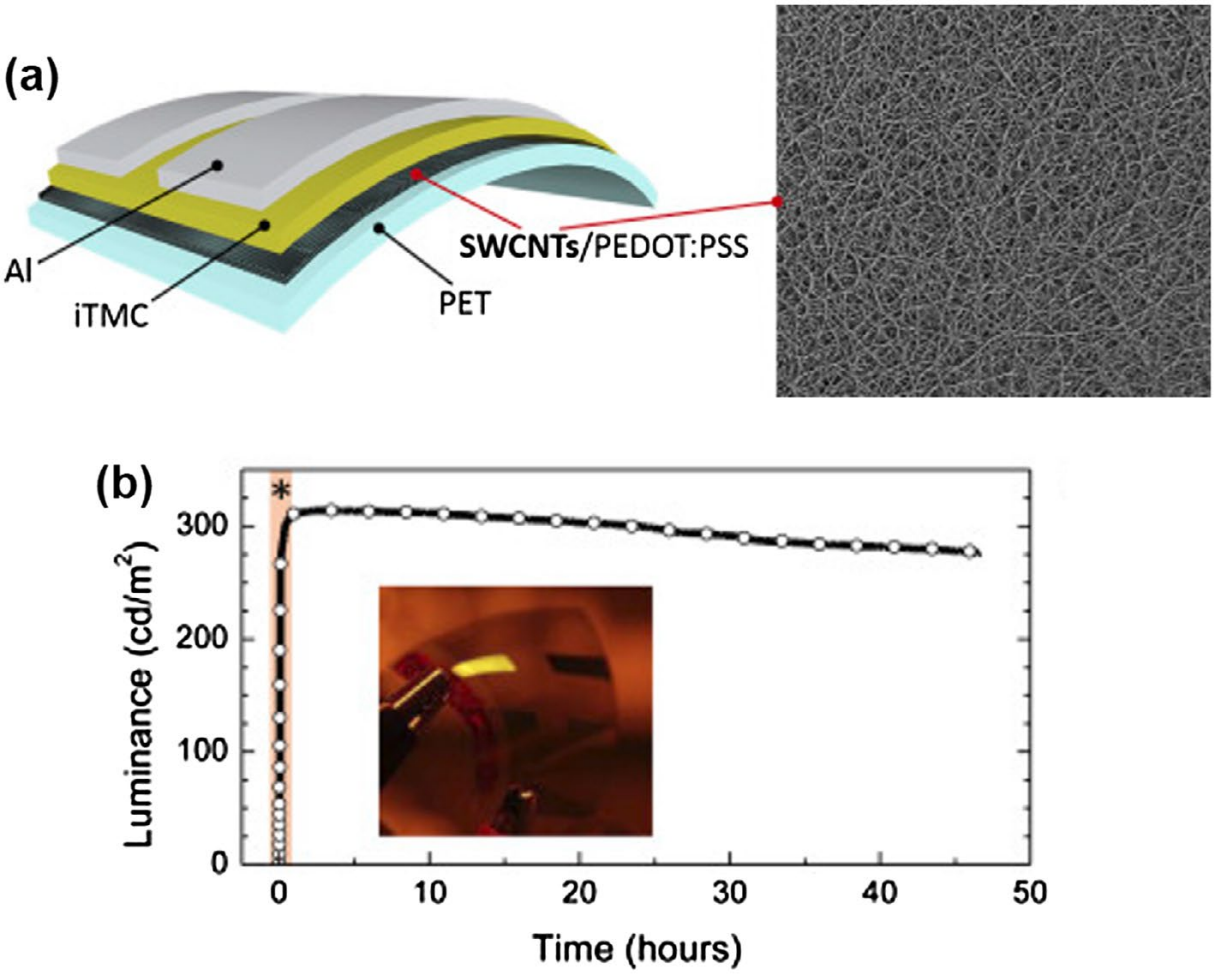
(a) Schematics of the flexible device layout built on the PET/CNTs/PEDOT:PSS conducting substrate and SEM image of CNT films; iTMC stands for ionic transition metal complex (b) Global luminance versus time. (Reprinted with permission from [186], copyright 2016 Elsevier.)

CNT-based TCFs enable easy fabrication of OLED and related devices on a plastic substrate. Martinez-Sarti [186] reported a flexible light-emitting electrochemical cell using CNT-based TCFs as an anode, as shown in Figure 13. The CNTs were synthesized by an integrated aerosol method, and then dry-transferred on to the plastic substrates. Deposition of a doped PEDOT:PSS film on the nanostructured CNT further homogenized the surface and enlarged the work function, which was expected to enhance the hole injection effects. By using an efficient phosphorescent ionic transition metal complex as a light emitting material, they achieved an efficacy up to 9 cd/A.

**Figure 14.**
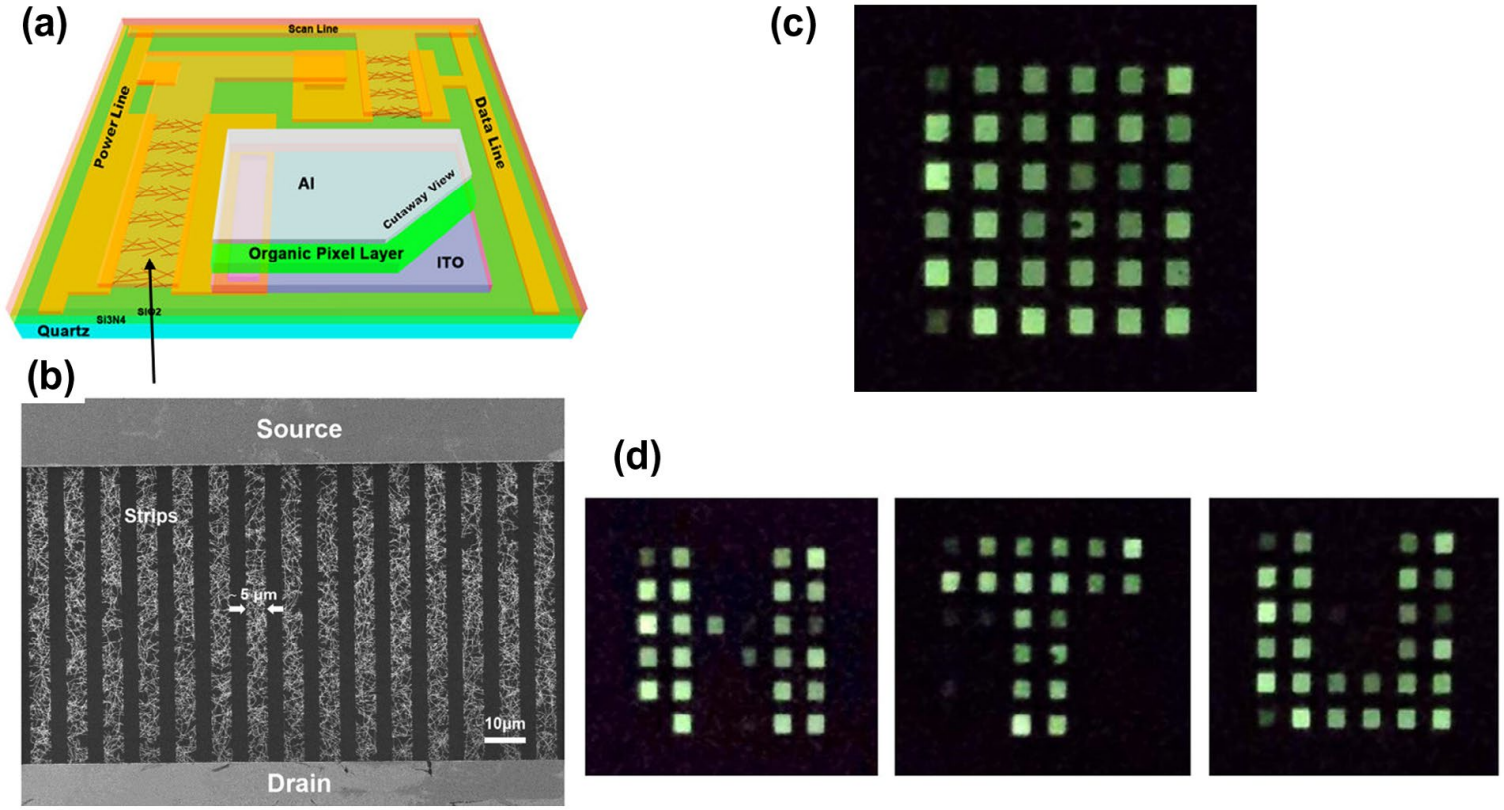
(a) Schematic view of OLED display. (b) SEM image of parallel CNT strips with a width of 5 μm. (c) A photo showing the 36 pixels. (d) The letters “N”, “T”, and “U” are displayed sequentially on this OLED display. (Reprinted with permission from [210], copyright 2015 Nature Publishing Group.)

Besides acting as a transparent electrode, CNT films can act as a hole injection layer to enhance the device efficiency, and also as a transistor to drive OLED displays.[195–210] OLED displays can use either passive-matrix (PMOLED) or active-matrix (AMOLED) addressing detailed schemes. AMOLED requires a thin-film transistor to not only switch each individual pixel on or off, but also allow higher resolution and larger display sizes. Transistors using semiconducting CNTs can drive AMOLED pixels at low operating voltage. Zou et al. [210] reported CVD-grown CNT network thin film transistor (TFT) driver circuits for static and dynamic AM OLED displays with 6  ×  6 pixels, as shown in Figure 14. The high device mobility of ~45 cm^2^ V^−1^s^−1^ and the high channel current on/off ratio of ~10^5^ of the CNT-TFTs enabled good control of the OLED pixels. The results suggest that CNT-TFTs are promising backplane building blocks for future OLED displays.

These various applications make CNT one of the most promising materials in OLED devices. CNT-TFTs exhibit higher mobility, channel current and longer lifetime than other TFTs using either silicon or organic semiconductors. Combination with CNT-based TCFs will significantly simplify the process for OLED displays. However, both of CNT-TFTs and CNT-based TCFs require easy but stable doping to improve or modify the electronic properties, which is also a main challenge for various devices using CNT thin films.

## 5. Summary and prospective

CNTs are allotropes of carbon with a one-dimensional cylindrical nanostructure. These one-dimensional carbon molecules have many outstanding properties, which have attracted much interest in various fields of materials science and technology. CNTs have been studied for over 25 years, but only during last few years have practical applications begun. Currently, the price of commercial, research grade CNT ranges from less than 1 US dollar per gram (multi-walled CNT) to hundreds of dollars per gram (single-walled CNT). The commercialization of CNTs, which have become cheaper and cheaper, is driving their practical application at industrial scale. More and more companies are making an attempt to introduce CNTs to upgrade their products. TCFs are a very promising field for CNTs, because of the rapid growth and development of customer electronics. According to [211] the market for TCFs will reach 1.2 billion dollars in 2025. Although ITO films will still dominate various applications of TCFs for a long period in the future, ITO alternative technologies such as metal nanowires and metal mesh are expected to expand their share in the coming years. CNT-based TCFs have to face a serious competition with ITO and alternative technologies.

It has been theoretically and experimentally proved that CNTs have a high intrinsic electrical conductivity close to metal, but it remains a challenge to transfer such high conductivity of individual CNTs to a network (film). Honghai and Canatu, the first two companies to manufacture CNT-based TCFs, have been developing dry processes to directly transfer their special CNTs onto substrate. They can manufacture high-quality CNT-based TCFs simply in mass production with a low-cost process. Many other studies in both academia and industry are using wet processes to fabricate CNT-based TCFs. The wet process is also promising since it not only allows the use of commercial CNTs with different qualities and prices, but also enables various deposition methods for desired applications. General wet processes contain several steps to deposit CNT films. Firstly, CNT aggregates with strong van der Waals binding energies should be dispersed or mixed in a solution. An ideal dispersion technique uses a small amount of environment-friendly dispersant, which should not degrade the conductivity of as-deposited CNT films. This technique should also supply an energy density between the binding energy of the aggregates, and the fracture resistance of individual CNTs, in order to minimize the damage to CNTs during dispersion. Unfortunately, lack of a feasible dispersion technology seriously suppresses the industrial use of wet-processed CNT films. Moreover, to obtain low sheet resistance of CNT, films need further modification/doping treatments. Although CNT-based TCFs have shown very promising performances such as 60 Ω/square at 90% transmittance in literature, most, using volatile dopants, are not stable. Doping with MoO_*x*_ or copper halides may be a breakthrough, while the use of high-temperature annealing or photonic curing processes greatly increases the production cost. Considering the performance/price competition among various TCFs, current technology of CNT-based TCFs is not optimistic.

The applications of CNT-based TCFs have been widely investigated in past decade. Sensing devices are the most promising field among various applications. Honghai and Canatu are manufacturing touch panels for mobile electronics. Therefore, more and more products using CNT-based TCFs will appear in the market and our daily life. OLED may be another promising application. CNT thin films with different properties/qualities can be used as a TCF, a hole injection layer for improving the efficiency, and also a TFT for driving OLED display. This will drive the use of such multifunctional CNT thin films in OLED applications. On the other hand, as listed in Table 2, the OPV cells fabricated on metal nanowires or metal mesh exhibit much better performances than those on CNT-based TCFs. OPV application requires better conductivity and transparency.

**Figure 15.**
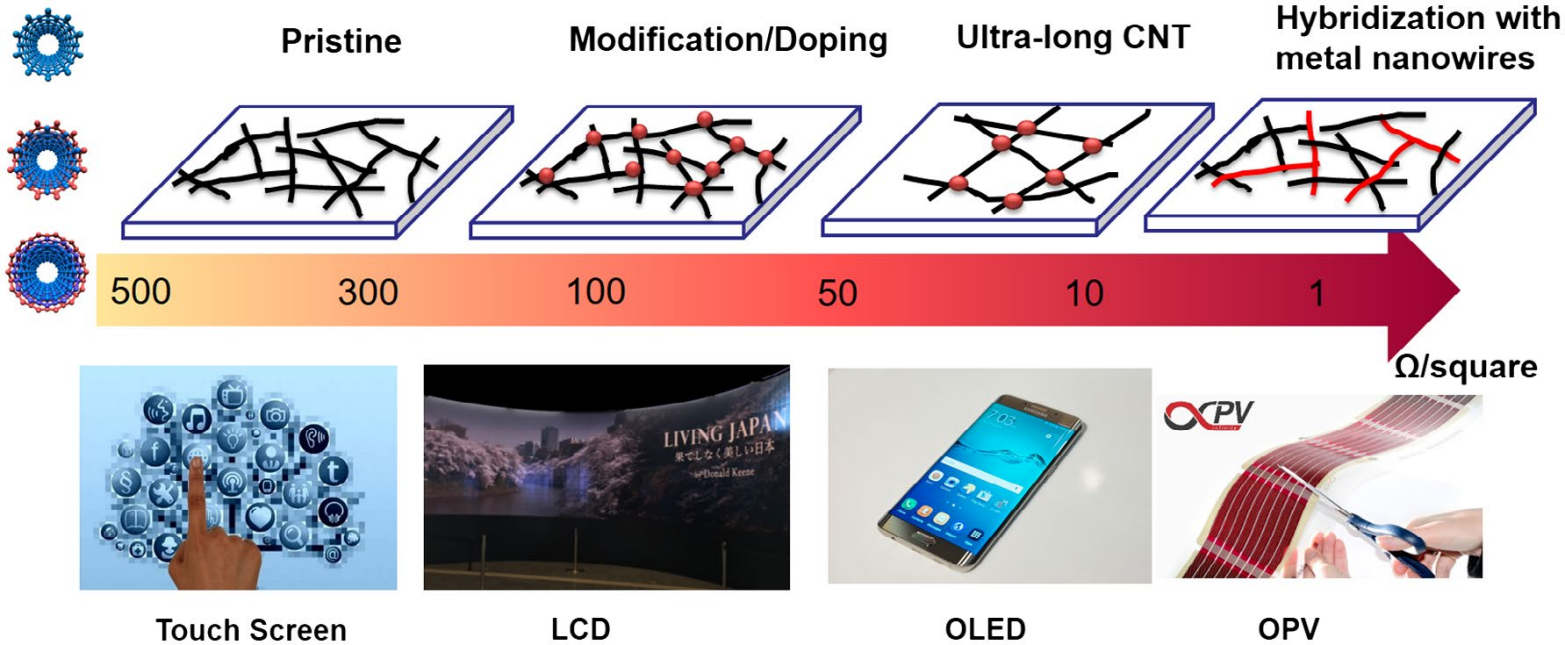
Various applications of CNT-based TCFs with a transmittance of above 85%. Pristine CNTs exhibit 300–500 Ω/square, while modification/doping will decrease the sheet resistance to 50–200 Ω/square. High-quality CNTs (i.e. ultra-long CNTs) may further reduce the sheet resistance to 10–50 Ω/square. Finally, incorporating with metal nanowires/nanoparticles can be efficient for boosting the conductivity for OPV applications. (The photographs of touch screen, OLED [213] and OPV [214] are reused via Google Advanced Image Search (free to use or share).)

Based on the overview of the fabrication, properties and possible applications of CNT-based TCFs, it can be concluded that current CNT-based TCFs still do not meet the demands of performance/cost for industrial use. There are two main challenges, which should be addressed in the coming years: decrease the produce cost and improve the conductivity and transparency. Despite the price of high-quality CNT (hundreds of dollars per gram) used for TCFs can be as low as 10 dollars per m^2^, the complex produce process is much more expensive than other TCFs. However, we still believe that the CNTs and CNT-based TCFs have a bright future. Figure 15 shows the possible applications of CNT-based TCFs. ITO is good enough for most applications. CNT-based TCFs should lead the applications among all the ITO alternatives. The conductivity of non-doped CNT-based TCFs with sheet resistances of 100–300 Ω/square at 90% transmittance meets the requirement of touch panels, while efforts for reducing the cost are needed. For example, multi-walled CNTs can be used. Moreover, developing a stable modification/doping will reduce the sheet resistance to 50–100 Ω/square, thus enabling liquid-crystal display (LCD) and OLED applications. Besides the inorganic semiconductors, organic molecules are expected to be stable and strong dopants. In particular, conducting polymers can also be used to disperse CNTs in solution. The development of organic molecules will push the revolution of fabrication process of CNTs. High-quality CNTs with a length of above 10 μm may further reduce the sheet resistance to 10–50 Ω/square. Current dispersion technology, using high-energy ultra-sonication, will unavoidably shorten the length of CNTs. Dispersing ultra-long CNTs with an aid of turbulent flow may also work for TCFs.[212] Finally, constructing a hybrid structure with CNTs and other high-conductivity materials can greatly improve the conductivity over the potential of CNTs for OPV applications. Strategically low price of commercial CNT-based TCFs will boost studies in both academia and industry, which will promote CNT-based TCFs in more and more products. Additionally, for the emerging flexible and wearable devices including sensing, lighting and energy harvesting devices, CNT-based TCFs can provide a platform which is more durable compared to other TCFs. TCFs are not a final product; many processes including high-temperature treatment, acid or alkaline treatment are further required. For example, for OLED displayers, TFTs using poly-silicon or oxides need a high temperature anneal above 200°C. Moreover, for hybrid perovskite solar cells, diffusion of iodine ions could erode the metal electrodes. In those cases, CNT-based TCFs will be a better choice rather than metal nanowire. On the other hand, CNT-based TCFs can be very stretchable and foldable. These special properties are motivating researchers of CNT-based TCFs to make further efforts to open a new field for future wearable optoelectronics.

## Funding

This work was supported by JSPS Grant-in-Aid for Research Activity Start-up (26888023) and Challenging Exploratory Research (16K14106).

## References

[CIT0001] Nathan A, Ahnood A, Cole MT (2012). Flexible electronics: the next ubiquitous platform. Proc IEEE.

[CIT0002] Kumar A, Zhou CW (2010). The race to replace tin-doped indium oxide: which material will win?. ACS Nano.

[CIT0003] Singh M, Haverinen HM, Dhagat P (2010). Inkjet printing-process and its applications. Adv Mater.

[CIT0004] Ellmer K (2012). Past achievements and future challenges in the development of optically transparent electrodes. Nat Photon.

[CIT0005] Song JZ, Zeng HB (2015). Transparent electrodes printed with nanocrystal inks for flexible smart dvices. Angew Chem Int Ed.

[CIT0006] Ginley DS, Bright C (2000). Transparent conducting oxides. MRS Bulletin.

[CIT0007] Minami T (2005). Transparent conducting oxide semiconductors for transparent electrodes. Semicond Sci Tech.

[CIT0008] Minami T (2008). Present status of transparent conducting oxide thin-film development for Indium-Tin-Oxide (ITO) substitutes. Thin Solid Film.

[CIT0009] Bühler G, Thölmann D, Feldmann C (2007). One-pot synthesis of highly conductive indium tin oxide nanocrystals. Adv Mater.

[CIT0010] Krebs FC, Gevorgyan SA, Alstrup J (2009). A roll-to-roll process to flexible polymer solar cells: model studies, manufacture and operational stability studies. J Mater Chem.

[CIT0011] Choulis SA, Choong VE, Mathai MK (2005). The effect of interfacial layer on the performance of organic light-emitting diodes. Appl Phys Lett.

[CIT0012] Vosgueritchian M, Lipomi DJ, Bao Z (2012). Highly conductive and transparent PEDOT: PSS films with a fluorosurfactant for stretchable and flexible transparent electrodes. Adv Funct Mater.

[CIT0013] Xia YJ, Sun K, Ouyang JY (2012). Solution-processed metallic conducting polymer films as transparent electrode of optoelectronic devices. Adv Mater.

[CIT0014] Alemu D, Wei HY, Ho KC (2012). Highly conductive PEDOT:PSS electrode by simple film treatment with methanol for ITO-free polymer solar cells. Energy Environ Sci.

[CIT0015] Vitoratos E, Sakkopoulos S, Dalas E (2009). Thermal degradation mechanisms of PEDOT:PSS. Org Electron.

[CIT0016] Wu H, Kong D, Ruan Z (2013). A transparent electrode based on a metal nanotrough network. Nat Nanotech.

[CIT0017] van de Groep JV, Spinelli P, Polman A (2012). Transparent conducting silver nanowire networks. Nano Lett.

[CIT0018] Ye SR, Rathmell AR, Chen ZF (2014). Metal nanowire networks: the next generation of transparent conductors. Adv Mater.

[CIT0019] Song M, You DS, Lim K (2013). Efficient and bendable organic solar cells with solution-processed silver nanowire electrodes. Adv Funct Mater.

[CIT0020] Ye SR, Stewart IE, Chen Z F (2016). How copper nanowires grow and how to control their properties. Acc. Chem. Res.

[CIT0021] Xu L, Yang Y, Hu ZW (2016). Comparison study on the stability of copper nanowires and their oxidation kinetics in gas and liquid. ACS Nano.

[CIT0022] Song J, Li J, Xu J (2014). Superstable transparent conductive Cu@Cu4Ni nanowire elastomer composites against oxidation, bending, stretching, and twisting for flexible and stretchable optoelectronics. Nano Lett.

[CIT0023] Bae S, Kim H, Lee Y (2010). Roll-to-roll production of 30-inch graphene films for transparent electrodes. Nat Nanotech.

[CIT0024] De S, Coleman JN (2010). Are there fundamental limitations on the sheet resistance and transmittance of thin graphene films?. ACS Nano.

[CIT0025] Kasry A, Kuroda MA, Martyna GJ (2010). Chemical doping of large-area stacked graphene films for use as transparent, conducting electrodes. ACS Nano.

[CIT0026] Wang SJ, Geng Y, Zheng QB (2010). Fabrication of highly conducting and transparent graphene films. Carbon.

[CIT0027] Güneş F, Shin HJ, Biswas C. (2010). Layer-by-layer doping of few-layer graphene film. ACS Nano.

[CIT0028] Park H, Brown PR, Buloyic V (2012). Graphene as transparent conducting electrodes in organic photovoltaics: studies in graphene morphology, hole transporting layers, and counter electrodes. Nano Lett.

[CIT0029] Jo G, Choe M, Lee S (2012). The application of graphene as electrodes in electrical and optical devices. Nanotech.

[CIT0030] Kim KS, Zhao Y, Jang H (2009). Large-scale pattern growth of graphene films for stretchable transparent electrodes. Nature.

[CIT0031] Khrapach I, Withers F, Bointon TH (2012). Novel highly conductive and transparent graphene-based conductors. Adv Mater.

[CIT0032] Jeong SY, Kim SH, Han JT (2011). High-performance transparent conductive films using rheologically derived reduced graphene oxide. ACS Nano.

[CIT0033] Park KH, Kim BH, Song SH (2012). Exfoliation of non-oxidized graphene flakes for scalable conductive film. Nano Lett.

[CIT0034] Pang S, Hernandez Y, Feng X (2011). Graphene as transparent electrode material for organic electronics. Adv Mater.

[CIT0035] Lee Y, Ahn JH (2013). Graphene-based transparent conductive films. Nano.

[CIT0036] Bu Q, Zhan YH, He FF (2016). Stretchable conductive films based on carbon nanomaterials prepared by spray coating. J Appl Polymer Sci.

[CIT0037] Hu L, Hecht DS, Gruner G (2004). Percolation in transparent and conducting carbon nanotube networks. Nano Lett.

[CIT0038] Hecht DS, Hu L, Gruner G (2011). Emerging transparent electrodes based on thin films of carbon nanotubes, graphene, and metallic nanostructures. Adv Mater.

[CIT0039] Geng HZ, Kim KK, So KP (2007). Effect of acid treatment on carbon nanotube-based flexible transparent conducting films. J Am Chem Soc.

[CIT0040] Hu L, Hecht DS, Gruner G (2010). Carbon nanotube thin films: fabrication, properties, and applications. Chem Rev.

[CIT0041] Dan B, Irvin GC, Pasquali M (2009). Continuous and scalable fabrication of transparent conducting carbon nanotube films. ACS Nano.

[CIT0042] Hou PX, Yu B, Su Y (2014). Double-wall carbon nanotube transparent conductive films with excellent performance. J Mater Chem A.

[CIT0043] Parekh BB, Fanchini G, Eda G (2007). Improved conductivity of transparent single-wall carbon nanotube thin films via stable postdeposition functionalization. Appl Phys Lett.

[CIT0044] Feng C, Liu K, Wu JS (2010). Flexible, stretchable, transparent conducting films made from superaligned carbon nanotubes. Adv Funct Mater.

[CIT0045] Doherty EM, De S, Lyons PE (2009). The spatial uniformity and electromechanical stability of transparent, conductive films of single walled nanotubes. Carbon.

[CIT0046] Simien D, Fagan JA, Luo W (2008). Influence of nanotube length on the optical and conductivity properties of thin single-wall carbon nanotube networks. ACS Nano.

[CIT0047] Yang SB, Kong BS, Jung DW (2011). Recent advances in hybrids of carbon nanotube network films and nanomaterials for their potential applications as transparent conducting films. Nanoscale.

[CIT0048] Du J, Pei S, Ma L (2014). Carbon nanotube- and graphene-based transparent conductive films for optoelectronic devices. Adv Mater.

[CIT0049] Ho XN, Wei J (2013). Films of carbon nanomaterials for transparent conductors. Materials.

[CIT0050] De Volder MFL, Tawfick SH, Baughman RH (2013). Carbon nanotubes: present and future commercial applications. Science.

[CIT0051] Wu Z1, Chen Z, Du X (2004). Transparent, conductive carbon nanotube films. Science.

[CIT0052] Saran N, Parikh K, Suh DS (2004). Fabrication and characterization of thin films of single-walled carbon nanotube bundles on flexible plastic substrates. J Am Chem Soc.

[CIT0053] Ma W, Song L, Yang R (2007). Directly synthesized strong, highly conducting, transparent single-walled carbon nanotube films. Nano Lett.

[CIT0054] Nasibulin AG, Kaskela A, Mustonen K (2011). Multifunctional free-standing single-walled carbon nanotube films. ACS Nano.

[CIT0055] Liu XL, Han S, Zhou CW (2006). Novel nanotube-on-insulator (NOI) approach toward single-walled carbon nanotube devices. Nano Lett.

[CIT0056] Gruner G (2006). Carbon nanotube films for transparent and plastic electronics. J Mater Chem.

[CIT0057] Zhang M, Fang S, Zakhidov AA (2005). Strong, transparent, multifunctional, carbon nanotube sheets. Science.

[CIT0058] Reynaud O, Nasibulin AG, Anisimov AS (2014). Aerosol feeding of catalyst precursor for CNT synthesis and highly conductive and transparent film fabrication. Chem Eng J.

[CIT0059] Cole M, Hiralal P, Ying K (2012). Dry-transfer of aligned multiwalled carbon nanotubes for flexible transparent thin films. J Nanomater.

[CIT0060] Chen J, Minett AI, Liu Y (2008). Direct growth of flexible carbon nanotube electrodes. Adv Mater.

[CIT0061] Kaskela A, Nasibulin AG, Timmermans MY (2010). Aerosol-synthesized SWCNT networks with tunable conductivity and transparency by a dry transfer technique. Nano Lett.

[CIT0062] http://www.canatu.com/canatu-launches-high-transmittance-cnb-transparent-conductive-film-for-touch-sensors/.

[CIT0063] Kato H, Nakamura A, Horie M (2014). Behavior of surfactants in aqueous dispersions of single-walled carbon nanotubes. RSC Adv.

[CIT0064] Xie XL, Mai YW, Zhou XP (2005). Dispersion and alignment of carbon nanotubes in polymer matrix: a review. Mater Sci Eng R.

[CIT0065] Vaisman L, Wagner HD, Marom G. (2006). The role of surfactants in dispersion of carbon nanotubes. Adv Colloid Interface Sci.

[CIT0066] Nish A, Hwang JY, Doig J (2007). Highly selective dispersion of single-walled carbon nanotubes using aromatic polymers. Nat Nanotech.

[CIT0067] Ausman KD, Piner R, Lourie O (2000). Organic solvent dispersions of single-walled carbon nanotubes: toward solutions of pristine nanotubes. J Phys Chem B.

[CIT0068] Rastogi R, Kaushal R, Tripathi SK (2008). Comparative study of carbon nanotube dispersion using surfactants. J Colloid Interface Sci.

[CIT0069] Kim SW, Kim T, Kim YS (2012). Surface modifications for the effective dispersion of carbon nanotubes in solvents and polymers. Carbon.

[CIT0070] Sun Z, Nicolosi V, Rickard D (2008). Quantitative evaluation of surfactant-stabilized single-walled carbon nanotubes: dispersion quality and its correlation with zeta potential. J Phys Chem C.

[CIT0071] Lee HW, Yoon Y, Park S (2011). Selective dispersion of high purity semiconducting single-walled carbon nanotubes with regioregular poly(3-alkylthiophene)s. Nat Commun.

[CIT0072] Huang YY, Terentjev EM (2012). Dispersion of Carbon Nanotubes: Mixing, Sonication, Stabilization, and Composite Properties. Polymer.

[CIT0073] Mistry KS, Larsen BA, Blackburn JL (2013). High-yield dispersions of large-diameter semiconducting single-walled carbon nanotubes with tunable narrow chirality distributions. ACS Nano.

[CIT0074] Fagan JA, Bauer BJ, Hobbie EK (2011). Carbon nanotubes: measuring dispersion and length. Adv Mater.

[CIT0075] Kharissova OV, Kharisov BI, Ortiz EGD (2013). Dispersion of carbon nanotubes in water and non-aqueous solvents. RSC Adv.

[CIT0076] Koh B, Park JB, Hou XM (2011). Comparative dispersion studies of single-walled carbon nanotubes in aqueous solution. J Phys Chem B.

[CIT0077] Barman SN, LeMieux MC, Baek J (2010). Effects of dispersion conditions of single-walled carbon nanotubes on the electrical characteristics of thin film network transistors. ACS Appl Mater Interfaces.

[CIT0078] Samanta SK, Fritsch M, Scherf U (2014). Conjugated polymer-assisted dispersion of single-wall carbon nanotubes: the power of polymer wrapping. Acc Chem Res.

[CIT0079] Rosner B, Guldi DM, Chen J (2014). Dispersion and characterization of arc discharge single-walled carbon nanotubes – towards conducting transparent films. Nanoscale.

[CIT0080] Yang SB, Kong BS, Kim DW (2010). Functionalizing single-walled carbon nanotube networks: effect on electrical and electrochemical properties. J Phys Chem C.

[CIT0081] Li Z, Kandel HR, Dervishi E (2007). Does the wall number of carbon nanotubes matter as conductive transparent material?. Appl Phys Lett.

[CIT0082] Zhang D, Ryu K, Liu X (2006). Transparent, conductive, and flexible carbon nanotube films and their application in organic light-emitting diodes. Nano Lett.

[CIT0083] Jo JW, Jung JW, Lee JU (2010). Fabrication of highly conductive and transparent thin films from single-walled carbon nanotubes using a new non-ionic surfactant via spin coating. ACS Nano.

[CIT0084] Liu WB, Pei S, Du J (2011). Additive-free dispersion of single-walled carbon nanotubes and its application for transparent conductivefilms. Adv Funct Mater.

[CIT0085] Karousis N, Tagmatarchis N, Tasis D (2010). Current progress on the chemical modification of carbon nanotubes. Chem Rev.

[CIT0086] Maiti UN, Lee WJ, Lee JW (2014). Chemically modified/doped carbon nanotubes & graphene for optimized nanostructures & nanodevices. Adv Mater.

[CIT0087] Bekyarova E, Sarkar S, Wang F (2013). Effect of covalent chemistry on the electronic structure and properties of carbon nanotubes and graphene. Acc Chem Res.

[CIT0088] Peng X, Wong SS (2009). Functional covalent chemistry of carbon nanotube surfaces. Adv Mater.

[CIT0089] Cola BA, Xu J, Cheng CR (2007). Photoacoustic characterization of carbon nanotube array thermal interfaces. J Appl Phys.

[CIT0090] Santini CA, Volodin A, Van Haesendonck C (2011). Carbon nanotube-carbon nanotube contacts as an alternative towards low resistance horizontal interconnects. Carbon.

[CIT0091] Topinka MA, Rowell MW, Goldhaber-Gordon D (2009). Charge transport in interpenetrating networks of semiconducting and metallic carbon nanotubes. Nano Lett.

[CIT0092] Lee RS, Kim HJ, Fischer JE (1997). Conductivity enhancement in single-walled carbon nanotube bundles doped with K and Br. Nature.

[CIT0093] Nirmalraj PN, Lyons PE, De S (2009). Electrical connectivity in single-walled carbon nanotube networks. Nano Lett.

[CIT0094] Kim Y, Chikamatsu M, Azumi R (2013). Industrially feasible approach to transparent, flexible, and conductive carbon nanotube films: cellulose-assisted film deposition followed by solution and photonic processing. Appl Phys Express.

[CIT0095] Wang Y, Di C, Liu Y (2008). Optimizing single-walled carbon nanotube films for applications in electroluminescent devices. Adv Mater.

[CIT0096] Kim KK, Bae JJ, Park HK (2008). Fermi level engineering of single-walled carbon nanotubes by AuCl_3_ doping. J Am Chem Soc.

[CIT0097] Hellstrom SL, Vosgueritchian M, Stoltenberg RM (2012). Strong and stable doping of carbon nanotubes and graphene by MoO_x_ for transparent electrodes. Nano Lett.

[CIT0098] Mistry KS, Larsen BA, Bergeson JD (2011). n-Type transparent conducting films of small molecule and polymer amine doped single-walled carbon nanotubes. ACS Nano.

[CIT0099] Blackburn JL, Barnes TM, Beard MC (2008). Transparent conductive single-walled carbon nanotube networks with precisely tunable ratios of semiconducting and metallic nanotubes. ACS Nano.

[CIT0100] Rinzler AG, Donoghue EP (2011). All the dope on nanotube films. ACS Nano.

[CIT0101] Shim D, Jung SH, Han SY (2011). Improvement of SWCNT transparent conductive films via transition metal doping. Chem Commun.

[CIT0102] Hecht DS, Heintz AM, Lee R (2011). High conductivity transparent carbon nanotube films deposited from superacid. Nanotechnology.

[CIT0103] Mirri F, Ma AWK, Hsu TT (2012). High-performance carbon nanotube transparent conductive films by scalable dip coating. ACS Nano.

[CIT0104] Zhou Y, Shimada S, Saito T (2015). Building interconnects in carbon nanotube networks with metal halides for transparent electrodes. Carbon.

[CIT0105] Lee J, Woo JY, Kim JT (2014). Synergistically enhanced stability of highly flexible silver nanowire/carbon nanotube hybrid transparent electrodes by plasmonic welding. ACS Appl Mater Interfaces.

[CIT0106] Peng LW, Feng Y, Lv P (2012). Transparent, conductive, and flexible multiwalled carbon nanotube/graphene hybrid electrodes with two three-dimensional microstructures. J Phys Chem C.

[CIT0107] Xin GQ, Hwang W, Kim N (2010). A graphene sheet exfoliated with microwave irradiation and interlinked by carbon nanotubes for high-performance transparent flexible electrodes. Nanotechnology.

[CIT0108] Liu YP, Jung E, Wang Y (2014). Quasi- freestanding” graphene- on- single walled carbon nanotube electrode for applications in organic light- emitting diode. Small.

[CIT0109] Stapleton AJ, Afre RA, Ellis AV (2013). Highly conductive interwoven carbon nanotube and silver nanowire transparent electrodes. Sci Technol Adv Mater.

[CIT0110] Stapleton AJ, Yambem SD, Johns AH (2015). Planar silver nanowire, carbon nanotube and PEDOT:PSS nanocomposite transparent electrodes. Sci Technol Adv Mater.

[CIT0111] Woo JY, Kim KK, Lee J (2014). Highly conductive and stretchable Ag nanowire/carbon nanotube hybrid conductors. Nanotechnology.

[CIT0112] Han HJ, Choi YC, Han JH (2015). Preparation of transparent conducting films with improved haze characteristics using single-wall carbon nanotube-silver nanowire hybrid material. Synt Met.

[CIT0113] Woo JS, Han JT, Jung S (2014). Electrically robust metal nanowire network formation by in-situ interconnection with single-walled carbon nanotubes. Sci Rep.

[CIT0114] Jing M, Han C, Li M (2014). High performance of carbon nanotubes/silver nanowires-PET hybrid flexible transparent conductive films via facile pressing-transfer technique. Nanoscale Res Lett.

[CIT0115] Kim D, Zhu L, Jeong DJ (2013). Transparent flexible heater based on hybrid of carbon nanotubes and silver nanowires. Carbon.

[CIT0116] Thostenson E, Li C, Chou T (2005). Nanocomposites in context. Composites Sci Tech.

[CIT0117] Jackson R, Domercq B, Jain R (2008). Stability of doped transparent carbon nanotube electrodes. Adv Funct Mater.

[CIT0118] Zhou Y, Shimada S, Saito T (2015). Understanding the doping effects on the structural and electrical properties of ultrathin carbon nanotube networks. J Appl Phys.

[CIT0119] Murase S, Yang Y (2012). Solution processed MoO_3_ interfacial layer for organic photovoltaics prepared by a facile synthesis method. Adv Mater.

[CIT0120] Zhou Y, Taima T, Miyadera T (2012). Glancing angle deposition of copper iodide nanocrystals for efficient organic photovoltaics. Nano Lett.

[CIT0121] Zhou Y, Taima T, Miyadera T (2012). Phase separation of co-evaporated ZnPc:C60 blend film for highly efficient organic photovoltaics. Appl Phys Lett.

[CIT0122] Walker G (2012). A review of technologies for sensing contact location on the surface of a display. J Soc Inf Disp.

[CIT0123] Chang-Jian SK, Ho JR (2011). John Cheng, JW. Fabrication of transparent double-walled carbon nanotubes flexible matrix touch panel by laser ablation technique. J W Opt Laser Technol.

[CIT0124] Park C, Kim SW, Lee YS (2012). Spray coating of carbon nanotube on polyethylene terephthalate film for touch panel application. J Nanosci Nanotechnol.

[CIT0125] Kim BJ, Han SH, Park JS (2014). Sheet resistance, transmittance, and chromatic property of CNTs coated with PEDOT:PSS films for transparent electrodes of touch screen panels. Thin Solid Film.

[CIT0126] Kim W, Oh H, Kwak Y (2015). Development of a carbon nanotube-based touchscreen capable of multi-touch and multi-force sensing. Sensor.

[CIT0127] Lee P, Ham J, Lee J (2014). Highly stretchable or transparent conductor fabrication by a hierarchical multiscale hybrid nanocomposite. Adv Funct Mater.

[CIT0128] Lee W, Koo H, Sun J (2015). A fully roll-to-roll gravure-printed carbon nanotube-based active matrix for multi-touch sensors. Sci Rep.

[CIT0129] Bai S, Sun C, Yan H (2015). Healable, transparent, room-temperature electronic sensors based on carbon nanotube network-coated polyelectrolyte multilayers. Small.

[CIT0130] Zhang X, Hu S, Wang M (2015). Continuous graphene and carbon nanotube based high flexible and transparent pressure sensor arrays. Nanotechnology.

[CIT0131] Cai L, Song L, Luan P (2013). Super-stretchable, transparent carbon nanotube-based capacitive strain sensors for human motion detection. Sci Rep.

[CIT0132] Lee KH, Scardaci V, Kim HY (2013). Highly sensitive, transparent, and flexible gas sensors based on gold nanoparticle decorated carbon nanotubes. Actuat B Chem.

[CIT0133] Lee S, Reuveny A, Reeder J (2016). A transparent bending-insensitive pressure sensor.

[CIT0134] Wang XL, Li TJ, Adam J (2013). Transparent, stretchable, carbon-nanotube-inlaid conductors enabled by standard replication technology for capacitive pressure, strain and touch sensors. J Mater Chem A.

[CIT0135] Nossol E, Zarbin AJG (2012). Transparent films from carbon nanotubes/Prussian blue nanocomposites: preparation, characterization, and application as electrochemical sensors. J Mater Chem.

[CIT0136] Choi E, Kim J, Chun S (2011). Fabrication of a flexible and transparent touch sensor using single-walled carbon nanotube thin-films. J Nanosci Nanotechnol.

[CIT0137] Lipomi DJ, Vosgueritchian M, Tee BCK (2011). Skin-like pressure and strain sensors based on transparent elastic films of carbon nanotubes. Nat Nanotechnol.

[CIT0138] Kuila BK, Stamm M (2010). Transparent, versatile chemical vapor sensor using supramolecular assembly of block copolymer and carbon nanotubes. Macromol Rapid Commun.

[CIT0139] Cohen DJ, Mitra D, Peterson K (2012). A highly elastic, capacitive strain gauge based on percolating nanotube networks. Nano Lett.

[CIT0140] Liu K, Sun YH, Liu P (2011). Cross-stacked superaligned carbon nanotube films for transparent and stretchable conductors. Adv Funct Mater.

[CIT0141] Muth JT, Vogt DM, Truby RL (2012). Embedded 3D printing of strain sensors within highly stretchable elastomers. Adv Mater.

[CIT0142] Saha A, Jiang C, Marti AA (2014). Carbon nanotube networks on different platforms. Carbon.

[CIT0143] Kim BS, Lee SW, Yoon H (2010). Pattern transfer printing of multiwalled carbon nanotube multilayers and application in biosensors. Chem Mater.

[CIT0144] Roh E, Hwang BU, Kim D (2015). Stretchable, transparent, ultrasensitive, and patchable strain sensor for human-machine interfaces comprising a nanohybrid of carbon nanotubes and conductive elastomers. ACS Nano.

[CIT0145] Tenent RC, Barnes TM, Bergeson JD (2009). Ultrasmooth, large-area, high-uniformity, conductive transparent single-walled-carbon-nanotube films for photovoltaics produced by ultrasonic spraying. Adv Mater.

[CIT0146] Kim S, Yim J, Wang X (2010). Spin- and spray-deposited single-walled carbon-nanotube electrodes for organic solar cells. Adv Funct Mater.

[CIT0147] Barnes TM, Bergeson JD, Tenent RC (2010). Carbon nanotube network electrodes enabling efficient organic solar cells without a hole transport layer. Appl Phys Lett.

[CIT0148] Tyler TP, Brock RE, Karmel HJ (2011). Electronically monodisperse single-walled carbon nanotube thin films as transparent conducting anodes in organic photovoltaic devices. Adv Energy Mater.

[CIT0149] Salvatierra RV, Cava CE, Roman LS (2013). ITO-free and flexible organic photovoltaic device based on high transparent and conductive polyaniline/carbon nanotube thin films. Adv Funct Mater.

[CIT0150] Rowell MW, Topinka MA, McGehee MD (2006). Organic solar cells with carbon nanotube network electrodes. Appl Phys Lett.

[CIT0151] Hu X, Chen L, Zhang Y (2014). Large-scale roll-to-roll fabrication of ordered mesoporous materials using resol-assisted cooperative assembly. Chem Mater.

[CIT0152] Jeon I, Cui K, Chiba T (2015). Direct and dry deposited single-walled carbon nanotube films doped with MoO_x_ as electron-blocking transparent electrodes for flexible organic solar cells. J Am Chem Soc.

[CIT0153] Zhou Y, Wang Z, Saito T (2016). Fabrication of carbon nanotube hybrid films as transparent electrodes for small-molecule photovoltaic cell. RSC Adv.

[CIT0154] Cataldo S, Salice P, Menna E (2012). Carbon nanotubes and organic solar cells. Energy Environ Sci.

[CIT0155] Su CY, Lu AY, Chen YL (2010). Chemically-treated single-walled carbon nanotubes as digitated penetrating electrodes in organic solar cells. J Mater Chem.

[CIT0156] Cho DY, Eun K, Choa SH (2014). Highly flexible and stretchable carbon nanotube network electrodes prepared by simple brush painting for cost-effective flexible organic solar cells. Carbon.

[CIT0157] Kim S, Wang X, Yim JH (2012). Efficient organic solar cells based on spray-patterned single wall carbon nanotube electrodes. J Photon Energy.

[CIT0158] Keru G, Ndungu PG, Nyamori VO (2014). A review on carbon nanotube/polymer composites for organic solar cells. Int J Energy Res.

[CIT0159] Lipomi D, Bao Z (2011). Stretchable, elastic materials and devices for solar energy conversion. Energy Environ Sci.

[CIT0160] Angmo D, Krebs FC (2013). Flexible ITO-free polymer solar cells. J Appl Polymer Sci.

[CIT0161] Su DS, Centi G (2013). A perspective on carbon materials for future energy application. J Energy Chem.

[CIT0162] van de Lagemaat J, Barnes TM, Rumbles G (2006). Organic solar cells with carbon nanotubes replacing In2O3: Sn as the transparent electrode. Appl Phys Lett.

[CIT0163] Son SY, Yun JM, Noh YJ (2015). Highly flexible and bendable carbon nanosheets as transparent conducting electrodes for organic solar cells. Carbon.

[CIT0164] Feng YY, Ju X, Feng W (2009). Organic solar cells using few-walled carbon nanotubes electrode controlled by the balance between sheet resistance and the transparency. Appl Phys Lett.

[CIT0165] Jin SH, Jun GH, Hong SH (2012). Conformal coating of titanium suboxide on carbon nanotube networks by atomic layer deposition for inverted organic photovoltaic cells. Carbon.

[CIT0166] Zhou Y, Taima T, Kuwabara T (2013). Efficient small-molecule photovoltaic cells using a crystalline diindenoperylene film as a nanostructured template. Adv Mater.

[CIT0167] Jeon I, Chiba T, Delacou C (2015). Single-walled carbon nanotube film as electrode in indium-free planar heterojunction perovskite solar cells: investigation of electron-blocking layers and dopants. Nano Lett.

[CIT0168] Li Z, Kulkarni SA, Boix PP (2014). Laminated carbon nanotube networks for metal electrode-free efficient perovskite solar cells. ACS Nano.

[CIT0169] Sachse C, Weiss N, Gaponik N (2014). ITO-free, small-molecule organic solar cells on spray-coated copper-nanowire-based Transparent Electrodes. Adv Energy Mater.

[CIT0170] Kim Y, Ryu TI, Ok KH (2015). Inverted layer-by-layer fabrication of an ultraflexible and transparent Ag nanowire/conductive polymer composite electrode for use in high-performance organic solar cells. Adv Funct Mater.

[CIT0171] Chen KS, Yip HL, Salinas JF (2014). Strong photocurrent enhancements in highly efficient flexible organic solar cells by adopting a microcavity configuration. Adv Mater.

[CIT0172] Kang H, Jung S, Jeong S (2015). Polymer-metal hybrid transparent electrodes for flexible electronics. Nat Commun.

[CIT0173] Li Y, Meng L, Yang YM (2016). High-efficiency robust perovskite solar cells on ultrathin flexible substrates. Nat Commun.

[CIT0174] Li J, Hu L, Wang L (2006). Organic light-emitting diodes having carbon nanotube anodes. Nano Lett.

[CIT0175] Aguirre CM, Auvray S, Pigeon S (2006). Carbon nanotube sheets as electrodes in organic light-emitting diodes. Appl Phys Lett.

[CIT0176] Bansal M, Srivastava R, Lal C (2009). Carbon nanotube-based organic light emitting diodes. Nanoscale.

[CIT0177] Hu LB, Li JF, Liu J (2010). Flexible organic light-emitting diodes with transparent carbon nanotube electrodes: problems and solutions. Nanotechnology.

[CIT0178] Liu D, Fina M, Guo J (2009). Organic light-emitting diodes with carbon nanotube cathode-organic interface layer. Appl Phys Lett.

[CIT0179] Gao J, Mu X, Li XY (2013). Modification of carbon nanotube transparent conducting films for electrodes in organic light-emitting diodes. Nanotechnology.

[CIT0180] Yu Z, Hu L, Liu Z (2009). Fully bendable polymer light emitting devices with carbon nanotubes as cathode and anode. Appl Phys Lett.

[CIT0181] Xu F, Zhu WQ, Yan L (2012). Single walled carbon nanotube anodes based high performance organic light-emitting diodes with enhanced contrast ratio. Org Electron.

[CIT0182] Kim M, Kim YC (2014). Single wall carbon nanotube/poly (3, 4-ethylenedioxythiophene) nanocomposite film as a transparent electrode for flexible organic light-emitting diodes. Synt Met.

[CIT0183] Zhang B, Li F, Lin Z (2012). Flexible white organic light-emitting diodes based on single-walled carbon nanotube:poly(3,4-ethylenedioxythiophene)/poly(styrene sulfonate) transparent conducting film. Jpn. J Appl Phys.

[CIT0184] Sam FLM, Dabera GDMR, Lai KT (2014). Hybrid metal grid-polymer-carbon nanotube electrodes for high luminance organic light emitting diodes. Nanotechnology.

[CIT0185] Freitag P, Zakhidov AA, Luessem B (2012). Lambertian white top-emitting organic light emitting device with carbon nanotube cathode. J Appl Phys.

[CIT0186] Martínez-Sarti L, Pertegás A, Monrabal-Capilla M (2016). Flexible light-emitting electrochemical cells with single-walled carbon nanotube anodes. Org Electron.

[CIT0187] Inigo AR, Underwood JM, Silva SRP (2011). Carbon nanotube modified electrodes for enhanced brightness in organic light emitting devices. Carbon.

[CIT0188] Williams CD, Robles RO, Zhang M (2008). Multiwalled carbon nanotube sheets as transparent electrodes in high brightness organic light-emitting diodes. Appl Phys Lett.

[CIT0189] Chhowalla M (2007). Transparent and conducting SWNT thin films for flexible electronics. J Soc Inf Disp.

[CIT0190] Qu ECW, Hu L, Raymond GCR (2009). Surface-modified nanotube anodes for high performance organic light-emitting diode. ACS Nano.

[CIT0191] Yu Z, Liu Z, Wang M (2011). Highly flexible polymer light-emitting devices using carbon nanotubes as both anodes and cathodes. J Photon Energy.

[CIT0192] Han TH, Jeong SH, Lee Y (2015). Flexible transparent electrodes for organic light-emitting diodes. J Inf Disp.

[CIT0193] Xu J, Smith GM, Dun C (2015). Layered, nanonetwork composite cathodes for flexible, high-efficiency, organic light emitting devices. Adv Funct Mater.

[CIT0194] Zhong C, Duan C, Huang F (2011). Materials and devices toward fully solution processable organic light-emitting diodes. Chem Mater.

[CIT0195] Wang GF, Tao XM, Chen W (2007). Improvement in performance of organic light-emitting devices by inclusion of multi-wall carbon nanotubes. J Lumin.

[CIT0196] Zhang J, Wang C, Zhou C (2012). Rigid/flexible transparent electronics based on separated carbon nanotube thin-film transistors and their application in display electronics. ACS Nano.

[CIT0197] Xu W, Zhao J, Qian L (2014). Sorting of large-diameter semiconducting carbon nanotube and printed flexible driving circuit for organic light emitting diode (OLED). Nanoscale.

[CIT0198] Mills CA, Sam FLM, Alshammari AS (2014). Storage lifetime of polymer-carbon nanotube inks for use as charge transport layers in organic light emitting diodes. J Disp Technol.

[CIT0199] Shi S, Silva SRP (2012). High luminance organic light-emitting diodes with efficient multi-walled carbon nanotube hole injectors. Carbon.

[CIT0200] Chun JY, Han JW, Kim TW (2012). Enhancement of organic light-emitting diodes efficiency using carbon nanotube doped hole-injection layer on the Al-doped ZnO anode. ECS Solid State Lett.

[CIT0201] Zhang J, Fu Y, Wang C (2011). Separated carbon nanotube macroelectronics for active matrix organic light-emitting diode displays. Nano Lett.

[CIT0202] Gao L, Zhao S, Xu Z (2011). Effects of the introduced single-wall carbon nanotubes on the performance of blue phosphorescence organic light-emitting diodes. J Nanosci Nanotech.

[CIT0203] Shao M, Garrett MP, Xu XJ (2011). Effects of single walled carbon nanotubes on the electroluminescent performance of organic light-emitting diodes. Org Electron.

[CIT0204] McCarthy MA, Liu B, Donoghue EP (2011). Low-voltage, low-power, organic light-emitting transistors for active matrix displays. Science.

[CIT0205] Wang C, Zhang JL, Ryu KM (2009). Wafer-scale fabrication of separated carbon nanotube thin-film transistors for display applications. Nano Lett.

[CIT0206] Sekitani T, Nakajima H, Maeda H (2009). Stretchable active-matrix organic light-emitting diode display using printable elastic conductors. Nat Mater.

[CIT0207] Hatton RA, Blanchard NP, Tan LW (2009). Oxidised carbon nanotubes as solution processable, high work function hole-extraction layers for organic solar cells. Org Electron.

[CIT0208] Li J, Hu L, Liu J (2008). Indium tin oxide modified transparent nanotube thin films as effective anodes for flexible organic light-emitting diodes. Appl Phys Lett.

[CIT0209] Hatton RA, Miller AJ, Silva SRP (2008). Carbon nanotubes: a multi-functional material for organic optoelectronics. J Mater Chem.

[CIT0210] Zou J, Zhang K, Li J (2015). Carbon nanotube driver circuit for 6×6 organic light emitting diode display. Sci Rep.

[CIT0211] Overview report of Transparent Conductive Films (TCF) 2016-2026: Forecasts, Markets, Technologies. http://www.idtechex.com/research/reports/transparent-conductive-films-tcf-2015-2025-forecasts-markets-technologies-000437.asp.

[CIT0212] Yoon H, Yamashita M, Ata S (2014). Controlling exfoliation in order to minimize damage during dispersion of long SWCNTs for advanced composites. Sci Rep.

[CIT0213] https://commons.wikimedia.org/wiki/File:Samsung_Galaxy_S6_edge%2B.jpg.

[CIT0214] http://infinitypv.com/infinitypro/opv/foil.

